# Effects of an academic detailing service on benzodiazepine prescribing patterns in primary care

**DOI:** 10.1371/journal.pone.0289147

**Published:** 2023-07-27

**Authors:** Meagan Lacroix, Fred Abdelmalek, Karl Everett, Lena Salach, Lindsay Bevan, Victoria Burton, Noah M. Ivers, Mina Tadrous

**Affiliations:** 1 Women’s College Research Institute, Women’s College Hospital, Toronto, Ontario, Canada; 2 Temerty Faculty of Medicine, University of Toronto, Toronto, Ontario, Canada; 3 ICES, Toronto, Ontario, Canada; 4 Centre for Effective Practice, Toronto, Ontario, Canada; 5 University of Toronto, Toronto, Ontario, Canada; 6 Leslie Dan faculty of Pharmacy, University of Toronto, Toronto, Ontario, Canada; Royal College of Surgeons in Ireland, IRELAND

## Abstract

**Background:**

Benzodiazepines are commonly used to treat anxiety and/or insomnia but are associated with substantial safety risks. Changes to prescribing patterns in primary care may be facilitated through tailored quality improvement strategies. Academic detailing (AD) may be an effective method of promoting safe benzodiazepine prescribing. The objective of this study was to evaluate the effectiveness of AD on benzodiazepine prescribing among family physicians.

**Methods and findings:**

We used an interrupted time series matched cohort design using population-based administrative claims databases. Participants were family physicians practicing in Ontario, Canada. The intervention was a voluntary AD service which involves brief service-oriented educational outreach visits by a trained pharmacist. The focus was on key messages for safer benzodiazepine prescribing in primary care with an emphasis on judicious prescribing to older adults aged 65 and older. Physicians in the intervention group were those who received at least one AD visit on benzodiazepine use between June 2019 and February 2020. Physicians in the control group were included if they did not receive an AD visit during the study period. Intervention physicians were matched to control physicians 1:4, on a variety of characteristics. Physicians were excluded if they had inactive billing or billing of less than 100 unique patient visits in the calendar year prior to the index date. The primary outcome was mean total benzodiazepine prescriptions at the level of the physician. Secondary outcomes were rate (per 100) of patients with long-term prescriptions, high-risk prescriptions, newly started prescriptions, and benzodiazepine-related patient harms. Data were analyzed using a repeated measures pre-post comparison with an intention-to-treat. Analyses were then stratified to focus on effects within higher-prescribing physicians.

There were 1337 physicians were included in the study; 237 who received AD and 1064 who did not. There was no significant change in benzodiazepine prescribing when considering all physicians in the intervention and matched control groups. Although not significant, a greater reduction in total benzodiazepine prescriptions was observed amongst the highest-volume prescribing physicians who received the intervention (% change in slope = -0.53, 95%CI = -2.34 to 1.30, p > .05).

The main limitation of our study was the voluntary nature of the AD intervention, which may have introduced a self-selection bias of physicians most open to changing their prescribing.

**Conclusion:**

This study suggests that future AD interventions should focus on physicians with the greatest room for improvement to their prescribing.

## Introduction

Benzodiazepines are a sedative medication class frequently prescribed in primary care to treat symptoms of anxiety or sleep disorders. Long-term use of benzodiazepines and concurrent use of benzodiazepines with opioids are associated with adverse events, particularly in older populations [1]. To mitigate risks of harms from this medication class, clinical guidelines from both Canada and abroad recommend that benzodiazepine therapy be prescribed for the shortest possible duration, co-prescriptions with opioids be avoided, and newly-initiated benzodiazepine therapy only be used after careful consideration of other options [2–5].

Despite these guidelines, recent evidence shows continued high-risk benzodiazepine prescribing to community-dwelling residents [6–8]. In 2019, approximately 1 in 18 residents of Ontario, Canada received a benzodiazepine prescription, with nearly one third of those prescriptions being for more than 29 days, and nearly one quarter co-occurring with an opioid prescription [6]. An increase in high-risk benzodiazepine prescribing has also been observed in the United States [7]. From 2003 to 2015, the rate of ambulatory visits in which a benzodiazepine was prescribed by a primary care physician increased from 3.6% to 7.5%. During this same time period, the rate of benzodiazepine and opioid co-prescriptions increased in the United States from 0.5% to 2% for outpatient visits. A recent UK study has found that in 2017, 44% of benzodiazepine prescriptions written by primary care physicians were longer than the recommended four weeks duration [8]. These studies highlight the difficulties of appropriate prescribing in primary care. Given that the majority of benzodiazepine prescriptions are written by primary care physicians, strategies aimed at improving benzodiazepine prescribing should focus on this group of prescribers [6,7].

Academic detailing (AD) is an educational outreach strategy which has been shown to improve prescribing in primary care [9,10]. This strategy involves a trained healthcare professional (e.g., a pharmacist) providing evidence-based information on best practices, including prescription medications, to a clinician in a 1-on-1 or small-group setting [11]. In Ontario, Canada, the Ministry of Health funds an AD initiative in primary care, which focused on benzodiazepine use in older adults from June 2019 to February 2020. The present study is an opportunistic, pragmatic evaluation of this initiative and its effects on benzodiazepine prescribing to a general patient population.

## Methods

The STROBE checklist (STrengthening the Reporting of OBservational studies in Epidemiology) was followed to ensure complete and accurate reporting of the methods used for this study.

### Design and setting

We conducted an interrupted time series analysis of benzodiazepine prescribing patterns among a matched cohort of family physicians practicing in Ontario. The intervention was an AD session on benzodiazepine use in older adults conducted between June 2019 and February 2020. Intervention participants were compared to a control group that did not receive an AD session. This study used population-based administrative data. Monthly data on benzodiazepine prescribing were collected over a period of 30 months: 12 months pre-and 18 months post-intervention.

In Ontario, Canada, most primary care is delivered by family physicians. While many family physicians work independently, some work in multidisciplinary Family Health Teams in which they may be supported by pharmacists and other health professionals. The provincial health insurance plan covers all medically necessary physician visits with co-pay and covers all prescription medications for patients on social assistance or aged 65 and above.

### Data sources

Outcomes were assessed using population-based administrative claims databases available at ICES (formerly known as the Institute for Clinical Evaluative Sciences). The ICES data repository consists of record-level, coded and linkable health data sets encompassing publicly funded administrative health services records from the Ontario population eligible for universal health coverage since 1986 [12]. Data collected by ICES initially contain personally identifiable information such as health card number, full name, and date of birth. This information is then replaced with a unique, confidential ICES Key Number (IKN), which ICES can then use to link information across data sets [13]. For the present study, outcomes were assessed using the following databases: (1) the Ontario Health Insurance Plan (OHIP) database to identify patients and their primary care providers; (2) the Ontario Drug Benefit (ODB) database to assess monthly prescriptions; (3) the Narcotics Monitoring System (NMS) database, which captures all opioid prescriptions dispensed in retail pharmacies across Ontario; (4) the Canadian Institute for Health Information (CIHI) database, which covers all inpatient hospitalizations and emergency department visits; (5) the Discharge Abstracts Database (DAD), National Ambulatory Care Reporting System (NACRS) databases to assign applicable diagnoses; (6) the Registered Persons Database (RPDB), which provides patient demographic information; (7) the ICES Physician Database (IPDB), which provides physician-specific details; and (8) the Drug Identification Number (DIN) database to identify the list of drugs from ODB formularies with DINs, generic and trade names, and strengths. Outcome data was linked with the list of CPSO numbers of the physicians who received an AD visit on this topic, and the date on which the AD visit occurred. The authors did not have access to any information which could potentially identify physicians or patients. Data received from ICES was in aggregate form only.

### Intervention

The AD service was provided by the Centre for Effective Practice (CEP), a not-for-profit organization focused on supporting the implementation of evidence-based practice [14]. The intervention involves tailored, brief educational outreach visits by a trained pharmacist (academic detailer). For this intervention, the focus was on key messages for safer benzodiazepine use in primary care with an emphasis on judicious prescribing to older adults aged 65 and older. Each participating family physician was offered a 1-on-1 visit of approximately 30-minutes in duration, with opportunity for follow-up to continue discussing the key messages and relevant resources (see https://cep.health/clinical-products/benzodiazepine-use-in-older-adults/ for details). CEP focuses on recruitment of providers in the regions of Ontario where an academic detailer is physically located, with recruitment efforts relying on snowball sampling. Academic detailers offered visits to family physicians who they had previously visited for other topics, as well as new family physicians who requested a visit after hearing about the service through word-of-mouth recruitment efforts. Visits were conducted in person by an academic detailer in the physician’s region. Engagement with the intervention was entirely voluntary. All physicians who volunteered to participate received an AD session unless they stopped responding to the academic detailer and were lost to follow up. The exact number of physicians lost to follow up is unknown, as CEP does not track that information. No financial incentives or accountability agreements existed to encourage participation, but physicians were eligible for one continuing medical education credit for each topic they received a visit on.

### Population and exposure

The intervention (AD) group consisted of family physicians actively practicing in the province of Ontario who voluntarily signed up and received at least one AD visit on benzodiazepine use in older adults between June 2019 and February 2020. Physicians were excluded if they had inactive billing or billing of less than 100 unique patient visits in the calendar year prior to the index date. This was to ensure a minimum practice level among physicians. The index date for physicians in the AD group was the date of their first AD visit on benzodiazepine prescribing.

Physicians in the matched control group were included if they never received an AD visit on any topic. We matched each physician in the AD group to four physicians in the matched control group on the following characteristics: index month, region (first letter of postal code), rate of benzodiazepine and opioid agonist therapy (OAT) prescribing in three months prior to index date (yes/no), sex (male/female), active status as an emergency room physician in three months prior to index date (yes/no), detailer is a Family Health Team member (yes/no) and number of years in practice at index (± 5). Quality checking of the resulting matches included comparing distributions of baseline variables for the two groups using standardized differences with a threshold of 0.1 or greater. Index date was randomly assigned to matched control physicians based on the frequency distribution of index month among AD physicians. See S1 Fig for a flow diagram of included physicians.

### Outcomes

While the academic detailing visit focuses on safer benzodiazepine prescribing to older adults, the principles of safer prescribing can also be applied to the general patient population as well. We chose to analyze total prescriptions to all patients to see if the intervention affected prescribing more broadly. The primary outcome was mean total benzodiazepine prescriptions at the level of the physician. This was measured as the total number of benzodiazepine prescriptions written by the physician and dispensed through the Narcotics Monitoring System. Secondary outcomes included long-term prescriptions, high-risk prescriptions, new start prescriptions, and benzodiazepine-related patient harms. Long-term benzodiazepine prescriptions were defined as 3 continuous 30-day prescriptions filled in a 100-day window. This definition is based on common dispensing patterns where prescriptions are written most often for 30 days [15,16]. High-risk prescriptions were defined as overlapping benzodiazepine and opioid prescriptions that put the patient at high risk for adverse outcomes, excluding patients with cancer or on palliative care. New-start prescriptions were defined as a benzodiazepine prescription to a patient who has not had a claim for this medication in the past year. Finally, benzodiazepine-related harms were measured as the number of benzodiazepine-related hospitalizations, ED visits, or falls. These were assessed using hospitalization codes in the DAD and NACRS databases. The codes used were ICD-10: T424, W00-W08, and W18-W19. Secondary outcomes were operationalized as rate (per 100) of patients per month with the occurrence, with the denominator being the number of unique patients with a benzodiazepine prescription in the same month.

### Statistical analysis

Descriptive statistics were calculated for all physicians and all patients who had a billing code from them during the three months prior to index. Continuous variables with a normal distribution were described using means and standard deviations, and categorical variables were summarized using frequencies and proportions. Analyses were repeated-measures with an intention-to-treat. The unit of analysis was the physician. All family physicians who received an AD visit on benzodiazepine prescribing between June 2019 and February 2020 and met the study inclusion criteria, together with their matched control providers, were included in the analysis.

Analyses of outcomes were planned *a priori* to be performed on the full sample of physicians based on all patient billings during each month of follow-up, as well as on two subgroups: physicians identified as being in the top 25% of benzodiazepine prescribing in the six months prior to index, and patients aged 65 and older. An additional sensitivity analysis was performed for physicians in the top 25% of benzodiazepine prescribing in the one month prior to index.

Outcomes were collected over 12 months pre-and 18 months post-index. To accommodate secular trends, outcomes for the control physicians were collected over the same duration relative to the index date as for their matched exposed physicians. To yield population-averaged estimates of effect, the primary and secondary outcomes were analyzed using generalized estimating equations (GEE) with a negative binomial family. To account for within-physician dependence of observations, models were fitted using GEE and an autoregressive correlation structure. The following regression model was fit to the data:

$$y_{t}\;=\;\beta_{0}+\beta_{1}G\;{+\;\beta }_{2}T\;{+\;\beta }_{3}TG_{\;}{+{\;\beta }_{4}TI_{\;}+\;\beta }_{5}T_{t-t_{post-index}}+\beta_{6}T{IG}_{\;}{+\;\beta }_{7}T_{t-t_{post-index}}G_{\;}+\;\epsilon_{t}$$

where $y_{t}$ is the measured numerator for each physician at time _t_, and $\beta_{0}$ represents the overall mean numerator at time = 0 (index) for physicians in the referent study group. The parameter $\beta_{1}$ indicates the mean difference in the numerator between two study groups at index. The pre-index slope (mean change in numerator per month) for the referent study group is given by ${\;\beta }_{2}$, and ${\;\beta }_{3}$ is the difference in pre-index slope between study group *G* compared to the referent group. The coefficients ${\;\beta }_{4}$ and ${\;\beta }_{5}$ respectively indicate the change in level and the change in slope between pre- to post-index for the referent study group. The differences in change in level and change in slope pre- to post-index for physicians in group *G* compared to the referent group are given by ${\;\beta }_{6}$ and ${\;\beta }_{7}$, respectively, with $\epsilon_{t}$ = the error term or residual. The natural log of the denominator was used as an offset.

Intervention effects are expressed as monthly percent change in slope together with robust 95% confidence intervals. All analyses were completed using SAS version 9.4 (SAS Institute) at ICES in Toronto, Ontario. Statistical significance was set at *p* < .05, and all tests were 2-tailed.

### Ethical considerations

Due to the observational nature of the study, a waiver was received from the Women’s College Hospital Research Ethics Board (REB) and no further ethical review was required. The REB determined that participants were not required to provide informed consent because a) the research involves no more than minimal risk to the participants; b) it would be impractical to carry out the research should consent be required; and c) all data provided to the study team was aggregated and fully anonymized. The study was reviewed by the ICES privacy department and all necessary data sharing agreements were obtained prior to conducting study activities.

## Results

A total of 1337 primary care physicians were included in the study, with 273 (20%) in the AD group and 1064 (80%) as matched controls. As seen in Table 1, physicians in the AD and matched control groups were well-balanced, although physicians in the AD group had a slightly smaller average roster size compared to the matched controls (1632.47 ± 920.05 vs. 1770.59 ± 1339.24). Details on types of benzodiazepines prescribed can be found in S1 Table in the appendix.

**Table 1:** Baseline characteristics of physicians and patients.

Physician Characteristics	No. (%)		
	Academic Detailing n = 273	Matched Controls n = 1064	Standardized Difference
Male	148 (54.2)	573 (53.9)	0.01
Age (mean, SE)	52.65 ± 10.91	52.33 ± 10.93	0.03
Years in practice (mean, SE)	22.90 ± 12.95	22.82 ± 12.86	0.01
Canadian medical graduate	170 (62.3)	653 (61.4)	0.02
Active as emergency room physician	38 (13.9)	130 (12.2)	0.05
Prescriber region			
Eastern Ontario	10 (3.7)	28 (2.6)	0.06
Central Ontario	83 (30.4)	332 (31.2)	0.02
Metropolitan Toronto	33 (12.1)	127 (11.9)	0.00
Southwestern Ontario	116 (42.5)	461 (43.3)	0.02
Northern Ontario	31 (11.4)	116 (10.9)	0.01
Urban location of primary practice	239 (87.5)	910 (85.5)	0.06
Roster size (mean, SE)	1632.47 ± 920.05	1770.59 ± 1339.24	0.12
Primary care model			
Enhanced fee for service	63 (23.1)	308 (28.9)	0.13
Non-team capitation	75 (27.5)	245 (23.0)	0.10
Family Health Team	167 (61.2)	643 (60.4)	0.02
Other	75 (27.5)	269 (25.3)	0.05
Multiple	83 (30.4)	303 (28.5)	0.04
Patient Characteristics (prior 3 months)	n = 193357	n = 765387	
Male	81649 (42.2)	325016 (42.5)	0.00
Age (mean, SE)	49.07 ± 24.42	46.81 ± 24.39	0.09
Income quintile			
1 (lowest)	37052 (19.2)	149269 (19.5)	0.01
2	37698 (19.5)	150012 (19.6)	0.00
3	39973 (20.7)	151991 (19.9)	0.02
4	39534 (20.4)	152168 (19.9)	0.01
5 (highest)	38371 (19.8)	158744 (20.7)	0.02
Cancer diagnosis	4059 (2.1)	14895 (1.9)	0.01
Palliative status	1418 (0.7)	5057 (0.7)	0.01
History of mental illness	43720 (22.6)	171856 (22.5)	0.00
Psychiatry consultation in year prior	10687 (5.5)	40864 (5.3)	0.01
Any benzodiazepine prescription	14244 (7.4)	55088 (7.2)	0.01

The subsample of physicians ranking in the top quartile of benzodiazepine prescribing included 67 physicians in the AD group and 266 physicians in the matched control group. These higher-prescribing groups were well-balanced on several characteristics (see Table 2), except for a slightly larger average roster size among AD group physicians (2065.13 ± 1239.32 vs 1849.95 ± 1548.84). Details on types of benzodiazepines prescribed by high prescribers can be found in S2 Table in the appendix.

**Table 2:** Baseline characteristics of physicians and patients–prescribers in top 25 percent.

Physician Characteristics	No. (%)		
	Academic Detailing n = 67	Matched Controls n = 266	Standardized Difference
Male	47 (70.1)	187 (70.3)	0.00
Age (mean, SE)	53.63 ± 11.55	54.44 ± 11.44	0.07
Years in practice (mean, SE)	25.57 ± 13.50	25.74 ± 13.37	0.01
Canadian medical graduate	39 (58.2)	161 (60.5)	0.05
Active as emergency room physician	< = 5* (7.5)	19 (7.1)	0.01
Prescriber region			
Eastern Ontario	< = 5* (1.5)	< = 5* (1.1)	0.03
Central Ontario	33 (49.3)	132 (44.6)	0.01
Metropolitan Toronto	6 (9.0)	24 (9.0)	0.00
Southwestern Ontario	20 (29.9)	79 (29.7)	0.00
Northern Ontario	7 (10.4)	28 (10.5)	0.00
Urban location of primary practice	61 (91.0)	232 (87.2)	0.12
Roster size (mean, SE)	2065.13 ± 1239.32	1849.95 ± 1548.84	0.15
Primary care model			
Enhanced fee for service	21 (31.3)	85 (32.0)	0.01
Non-team capitation	14 (20.9)	59 (22.2)	0.03
Family Health Team	40 (59.7)	158 (59.4)	0.01
Other	14 (20.9)	66 (24.8)	0.09
Multiple	15 (22.4)	74 (27.8)	0.13
Patient Characteristics (prior 3 months)	n = 63972	n = 200832	
Male	27774 (43.4)	89742 (44.7)	0.03
Age (mean, SE)	50.85 ± 24.28	47.53 ± 24.28	0.14
Income quintile			
1 (lowest)	14534 (22.7)	37638(18.7)	0.10
2	13724 (21.5)	38916 (19.4)	0.05
3	14331 (22.4)	42018 (20.9)	0.04
4	11448 (17.9)	40216 (20.0)	0.05
5 (highest)	9669 (15.1)	41257 (20.5)	0.14
Cancer diagnosis	1367 (2.1)	3789 (1.9)	0.02
Palliative status	623 (1.0)	1507(0.8)	0.02
History of mental illness	14956 (23.4)	44304 (22.1)	0.03
Psychiatry consultation in year prior	3894 (6.1)	9967 (5.0)	0.05
Any benzodiazepine prescription	6038 (9.4)	14679 (7.3)	0.08

* ICES’ *Protection of ICES Data* policy prohibits inclusion of small cells of fewer than five in any report or publication of the results of any ICES project or any research

Analyses were also repeated for a subsample of patients aged 65 and older. A total of 59,483 AD group patients and 206,853 matched control patients were included. Patient characteristics were again well-balanced. See S3 and S4 Tables in the appendix for details.

Between June 2019 and February 2020, physicians in the AD group received an average of 3.4 AD visits on any topic (including benzodiazepine use in older adults). Top prescribers received an average 3.8 AD visits on any topic. Details can be found in S5 Table in the appendix.

### Primary outcome: Total benzodiazepine prescriptions

**Fig 1:**
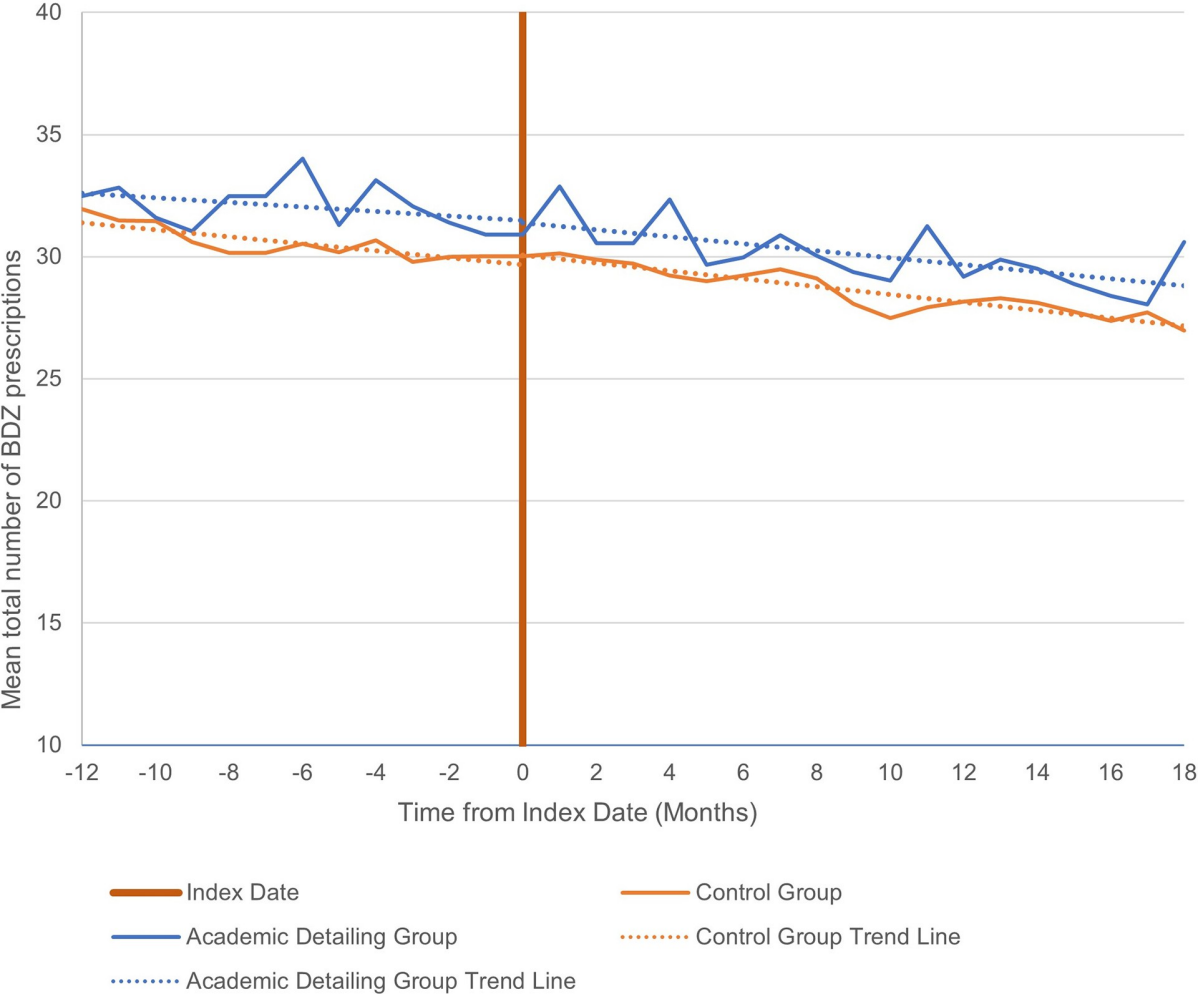
Mean total number of benzodiazepine prescriptions administered at 12 months pre- to 18 months post-intervention.

Over a 30-month period (12 months pre-intervention to 18 months post-intervention), the mean total number of benzodiazepine prescriptions per month decreased among physicians in both the AD group (32.47 to 30.61) and matched control group (31.95 to 26.28, see Fig 1). The decrease in total prescriptions for both groups began in the pre-intervention period, with matched controls showing a small but significant monthly percent change (-0.51, 95% CI = -0.92 to -0.10, *p* = .01), but not the AD group (-0.49, 95%CI = -1.21 to 0.23, *p* = .18). During the post-intervention period, neither group had a significant monthly percent change from pre-intervention and there were no significant differences between groups (see Table 3).

**Table 3:** Estimates of percent change in slope of total benzodiazepine prescriptions after the intervention vs before.

Total Prescriptions	Estimate (95% CI)	P-value
**All Physicians**		
AD group	0.00 (-0.82 to 0.83)	0.99
Matched Controls	-0.11 (-0.64 to 0.43)	0.69
% Difference (AD group vs Matched Controls)	0.11 (-0.87 to 1.10)	0.82
**Patients > 65**		
AD group	-0.10 (-1.00 to 0.81)	0.83
Matched Controls	-0.12 (-0.68 to 0.44)	0.67
% Difference (AD group vs Matched Controls)	0.01 (-0.87 to 0.91)	0.97
**Top Prescribers**		
AD group	-0.84 (-2.01 to 0.34)	0.16
Matched Controls	-0.31 (-1.69 to 1.09)	0.66
% Difference (AD group vs Matched Controls)	-0.53 (-2.34 to 1.30)	0.57

**Fig 2:**
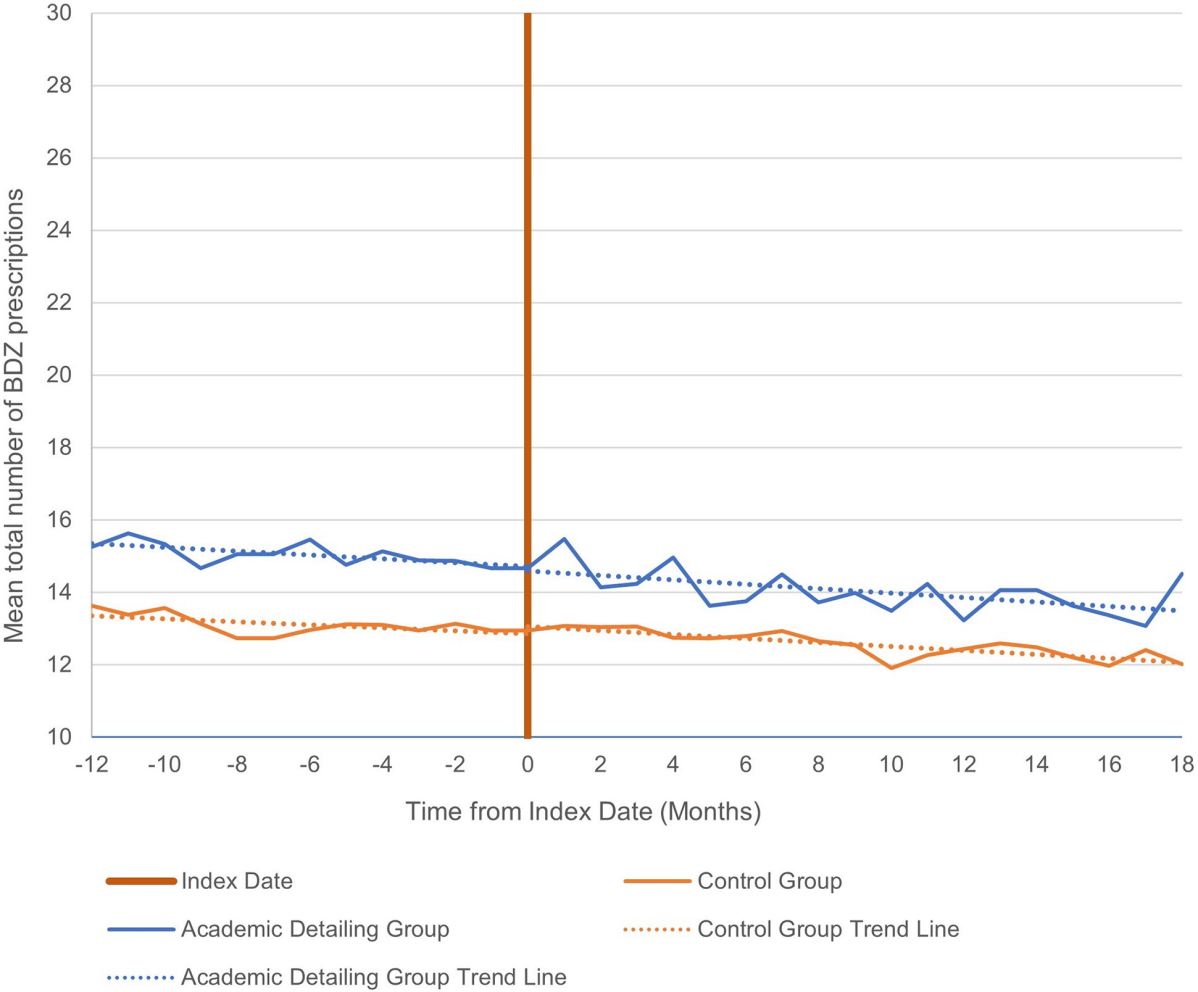
Mean total number of benzodiazepine prescriptions administered at 12 months pre- to 18 months post-intervention for patients > 65.

Similar trends in prescribing can be seen when limiting the data to only patients aged 65 and older (see Fig 2). Physicians in both groups showed a non-significant decrease in prescribing during the pre-intervention period, and this decrease continued into the post-intervention period, with no significant between-group differences (see Table 3).

**Fig 3:**
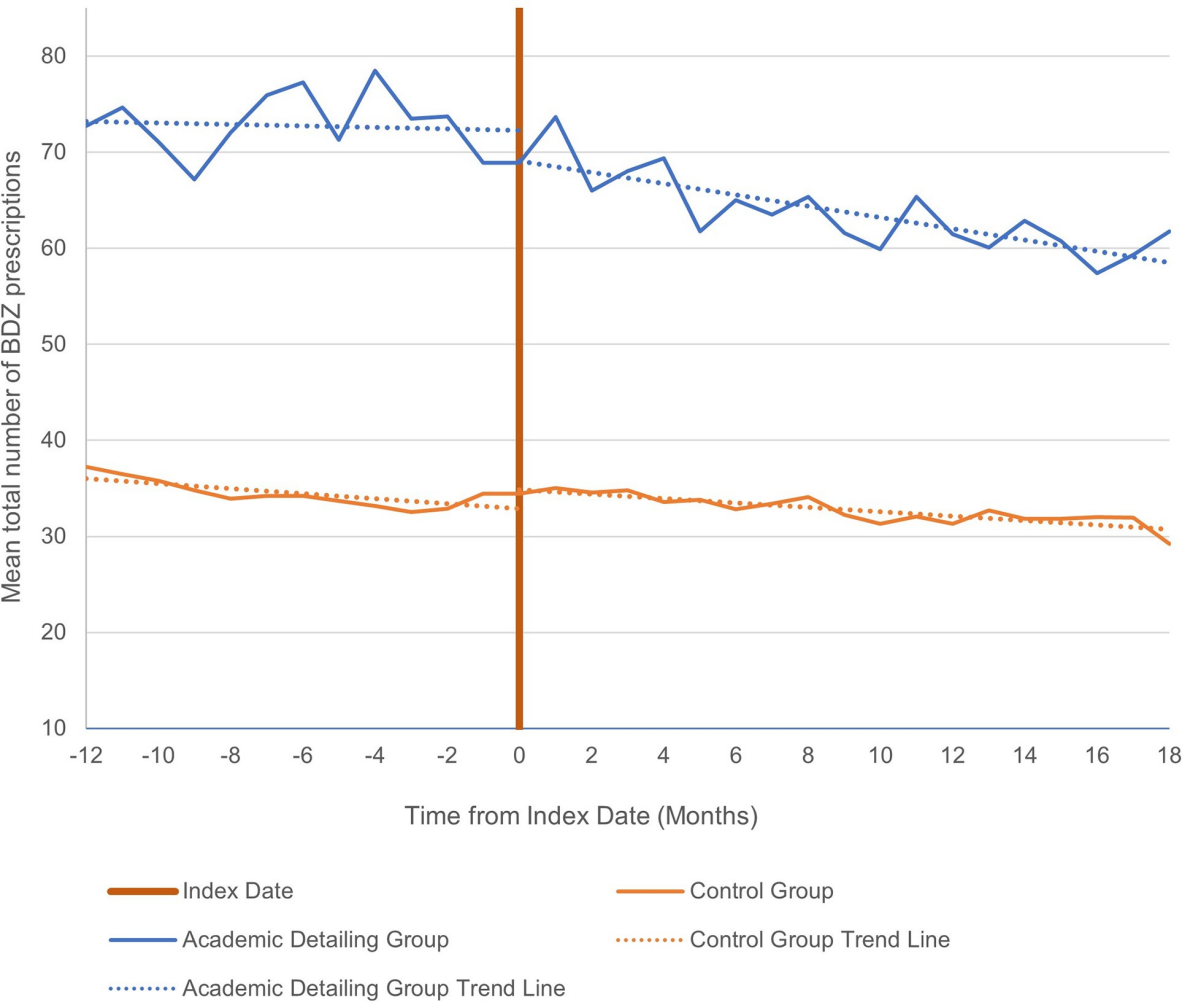
Mean total number of benzodiazepine prescriptions administered by top prescribers at 12 months pre- to 18 months post-intervention.

Over a 30-month period, the total number of prescriptions decreased among high-prescribing physicians in both the AD group (72.76 to 61.76) and matched controls (37.24 to 29.22, see Fig 3). It should be noted that differences in baseline prescribing rates between top prescribers in the AD group versus their matched controls is due to variations in the strength of the matching on the several characteristics outlined in the Population and Exposure section above. The percent change in slope from pre-intervention to 18-months post intervention was greater for the AD group compared to the matched controls, however this difference was not significant (see Table 3). Similar trends were seen in the sensitivity analysis (see S14 Fig).

### Secondary outcomes

There were no statistically significant differences between the AD group and matched control group for long-term benzodiazepine prescribing, co-prescribing with opioids, new-starts of benzodiazepines, or benzodiazepine-related harms. The percent change in slope increased for both the AD and matched control groups for long-term prescriptions, high-risk prescriptions, and benzodiazepine related harms. Similar results were found when limiting the data to patients ≥65. For top prescribing physicians, the percent change in slope increased for long-term and high-risk prescriptions, but decreased for benzodiazepine-related harms. For newly started prescriptions, the percent change in slope decreased for both groups overall, as well as when limiting the data to patients ≥65 and top prescribing physicians. See appendix for details.

## Discussion

In this quasi-experimental study, analyses indicate no significant overall effect of a real-world AD initiative on benzodiazepine prescribing. Overall, total monthly prescriptions and new prescriptions showed small decreases in the post-intervention period, while long-term prescriptions, high-risk prescriptions and benzodiazepine-related patient harms increased post-intervention. Interestingly, higher-prescribing physicians receiving AD showed a greater but non-significant reduction in their benzodiazepine prescribing compared to their matched controls. This suggests a potential opportunity to optimize the effects of an AD intervention by focusing the offering to those physicians with greatest room for improvement.

We observed small decreases in total prescriptions, long-term prescriptions, high-risk prescriptions, and benzodiazepine-related harms across all physicians in our study even prior to the intervention. This aligns with broader trends in prescribing in the province which found a 13% decrease in total benzodiazepine use among adults 35 and older from 2013–2019 [6], and a 2.5% decrease in opioid and benzodiazepine co-prescribing from 2013–2018 [17]. These downward secular trends may be related to an increased awareness of evidence-based guidelines highlighting the risks of benzodiazepines and approaches to dose reduction [2,18,19], as well as enhanced regulations such as the Narcotics Safety and Awareness Act in Ontario which allows the Ministry of Health to monitor the prescribing of controlled substances [20]. Additionally, alternative medications and non-pharmacological treatments for anxiety and sleep disorders have become more readily available [21]. The combination of these factors may help explain why no added benefit of AD was observed by many physicians in our study.

The results of this study highlight the difficulty of using AD to improve benzodiazepine prescribing in primary care. Past research has shown that AD can be an effective strategy for improving prescribing behavior of family physicians in a variety of settings and has been shown to be particularly effective for improving opioid prescribing [22,23]. The effectiveness of the CEP AD intervention in primary care was recently evaluated using pharmacy claims data. Researchers found that the CEP AD intervention led to a 37% improvement over a matched control group in reducing opioid doses, and a 58% improvement over matched controls in reducing high-risk opioid doses (i.e. morphine-equivalent doses >200mg/day) [22]. These findings were supported in another study which found that the CEP AD intervention led to a reduction in opioid prescribing compared to matched controls [23]. The effectiveness of AD on benzodiazepine prescribing has been less clear. In one systematic review of AD for prescribing, three studies focused on benzodiazepines [9]. Only one study [24] out of three [25,26] was successful in eliciting changes to prescribing behaviour. In the other two studies, physicians in the intervention group received only one 20-minute AD visit, while physicians in the successful study received two AD visits as well as a review of specific clinical cases. In another study aimed at reducing antibiotic prescribing, intervention group physicians received two educational outreach visits by a peer academic detailer, as well as a personalized report of prescribing data and a one-day educational seminar [27]. Intervention group physicians in this study showed a meaningful decrease in their antibiotic prescribing compared to controls. In a study looking at psychotropic prescribing in nursing homes, multidisciplinary teams in the intervention group who received monthly educational sessions over 12 months showed a significant decrease in their prescribing from baseline [28]. These educational sessions also included a review of the drug use of specific residents. The results from these studies may indicate that multiple AD visits—or AD visits lasting longer than 20 minutes—are more effective in eliciting prescribing changes for a particular medication class compared to a single AD visit. This may be especially important for making changes to complex prescribing behaviors such as those measured in this study. For the present study, the majority of clinicians in the intervention group received only a single AD visit on benzodiazepine prescribing. Only three clinicians received more than one AD visit on this topic. As seen in S5 Table, 37% of physicians in the AD group attended a total of 4 sessions on any topic. Future research on the effects of AD on benzodiazepine prescribing may benefit from comparing repeated to single intervention exposure as well as controlling for session length and exposure to other AD topics. Encouraging prescribers to review personal prescribing data or specific clinical cases may also maximize the effect of this educational intervention. The AD sessions provided by CEP do not include a targeted review of clinical cases. Including such a component may increase the effectiveness of the intervention.

The intervention group of our study was limited to physicians who volunteered to receive the AD intervention, which may have introduced a self-selection bias of physicians most open to changing their prescribing. One systematic review looking at the effectiveness of clinician-targeted education [29] found the greatest reduction in benzodiazepine prescribing in one study which did not rely on voluntary participation [30]. In this study, intervention group physicians were identified as any GP who prescribed to a Medicaid beneficiary deemed to be a “high user”. These physicians were mailed an educational package and showed a 27% reduction in benzodiazepine prescribing compared to the control group. The reductions seen in this study were greater than those seen in five other studies in which clinicians volunteered to receive face-to-face education [24–26,31,32]. In a more recent study, researchers targeted physicians identified as high prescribers of benzodiazepines to older adults [33]. These physicians were sent a personalized prescribing letter from the College of Physicians and Surgeons of Alberta (CPSA) identifying patients with high-risk benzodiazepine doses. Results showed a 50% reduction in number of patients receiving high-dose benzodiazepines as well as a 10% reduction in total benzodiazepine dosage. Though the researchers of this study were able to justify their methods of non-voluntary recruitment of high prescribers (i.e. CPSA has a legal obligation to intervene if public safety at risk), this may not be practical or feasible in a real-world setting where clinician education in the form of AD is encouraged but not forced.

The present study has limitations that warrant discussion. Given the retrospective, quasi-experimental design of this study, the data were not collected specifically for the study, but rather obtained through prescription databases. Patients not captured within the administrative databases (i.e. non-valid OHIP and prescriptions from non-primary care physicians) were excluded. It is possible that other effects may have been achieved in other safe-prescribing practices not easily measured through administrative data. For example, physicians may have improved their prescribing practice by discontinuing other medications which interact with benzodiazepines, or they may have improved patient education on the risks of benzodiazepines. While the pragmatic approach of this study may help shed light on the effectiveness of AD in real-world practice, it offers us less direct insight into the mechanism of action or on the efficacy of the intervention under more tightly controlled circumstances.

In summary, this quasi-experimental study evaluating a real-world intervention aiming to improve benzodiazepine prescribing in primary care found that a single AD visit was not associated with additional changes at a population level beyond those seen in secular trends. The voluntary nature of the intervention meant that some who engaged may have had little room for improvement. Those with greatest room for improvement in their prescribing behaviour seemed to benefit most. Despite these findings, past research has supported the use of AD as a means to improve appropriate prescribing of benzodiazepines. In the future, the effectiveness of AD may be enhanced by using a more targeted and data-driven approach to offering visits to physicians with room for improvement to their prescribing, as well as offering multiple or longer sessions and including a review of specific clinical cases.

## Supporting information

S1 Checklist STROBE Statement—Checklist of items that should be included in reports of observational studies. (DOCX)

S1 Fig Flow diagram of included AD physicians. (TIF)

S2 Fig Mean rate (per 100) of long-term benzodiazepine prescriptions at 12 months pre- to 18 months post-intervention. (TIF)

S3 Fig Mean rate (per 100) of long-term benzodiazepine prescriptions administered to patients >65 at 12 months pre- to 18 months post-intervention. (TIF)

S4 Fig Mean rate (per 100) of long-term benzodiazepine prescriptions administered by top prescribing physicians at 12 months pre- to 18 months post-intervention. (TIF)

S5 Fig Mean rate (per 100) of high-risk benzodiazepine prescriptions at 12 months pre- to 18 months post-intervention. (TIF)

S6 Fig Mean rate (per 100) of high-risk benzodiazepine prescriptions administered to patients >65 at 12 months pre- to 18 months post-intervention. (TIF)

S7 Fig Mean rate (per 100) of high-risk benzodiazepine prescriptions administered by top prescribing physicians at 12 months pre- to 18 months post-intervention. (TIF)

S8 Fig Mean rate (per 100) of new-start benzodiazepine prescriptions at 12 months pre- to 18 months post-intervention. (TIF)

S9 Fig Mean rate (per 100) of new-start benzodiazepine prescriptions administered to patients >65 at 12 months pre- to 18 months post-intervention. (TIF)

S10 Fig Mean rate (per 100) of new-start benzodiazepine prescriptions administered by top prescribing physicians at 12 months pre- to 18 months post-intervention. (TIF)

S11 Fig Mean rate (per 100) of patients experiencing benzodiazepine-related harms at 12 months pre- to 18 months post-intervention. (TIF)

S12 Fig Mean rate (per 100) of patients >65 experiencing benzodiazepine-related harms at 12 months pre- to 18 months post-intervention. (TIF)

S13 Fig Mean rate (per 100) of patients of top prescribers experiencing benzodiazepine -related harms at 12 months pre- to 18 months post-intervention. (TIF)

S14 Fig Mean total number of benzodiazepine prescriptions administered by top prescribers at 12 months pre- to 18 months post-intervention–sensitivity analysis. (TIF)

S15 Fig Mean rate (per 100) of long-term benzodiazepine prescriptions administered by top prescribing physicians at 12 months pre- to 18 months post-intervention–sensitivity analysis. (TIF)

S16 Fig Mean rate (per 100) of high-risk benzodiazepine prescriptions administered by top prescribing physicians at 12 months pre- to 18 months post-intervention–sensitivity analysis. (TIF)

S17 Fig Mean rate (per 100) of new-start benzodiazepine prescriptions administered by top prescribing physicians at 12 months pre- to 18 months post-intervention–sensitivity analysis. (TIF)

S18 Fig Mean rate (per 100) of patients of top prescribers experiencing benzodiazepine-related harms at 12 months pre- to 18 months post-intervention–sensitivity analysis. (TIF)

S1 Table Types of benzodiazepines received by patients. (PDF)

S2 Table Types of benzodiazepines received by patients of high prescribers. (PDF)

S3 Table Types of benzodiazepines received by patients >65. (PDF)

S4 Table Baseline characteristics of patients > 65 years. (PDF)

S5 Table. Number of academic detailing sessions attended on any topic (PDF)

S6 Table Estimates of percent change in slope of long-term benzodiazepine prescriptions after the intervention vs before. (PDF)

S7 Table Estimates of percent change in slope of high-risk benzodiazepine prescriptions after the intervention vs before. (PDF)

S8 Table Estimates of percent change in slope of new-start benzodiazepine prescriptions after the intervention vs before. (PDF)

S9 Table Estimates of percent change in slope of benzodiazepine-related patient harms after the intervention vs before. (PDF)

S10 Table Estimates of percent change in slope after the intervention vs before–sensitivity analysis for top prescribing physicians. (PDF)
