# Supplementary material for: Effects of an academic detailing service on benzodiazepine prescribing patterns in primary care
Source: PLoS One. 2023 Jul 27;18(7):e0289147. doi: 10.1371/journal.pone.0289147 (PMC10374092; doi:10.1371/journal.pone.0289147)
Supplement: S1 Checklist — (DOCX) [file pone.0289147.s001.docx]

STROBE Statement—checklist of items that should be included in reports of observational studies

|  | Item No. | Recommendation | Page  No. | Relevant text from manuscript |
| --- | --- | --- | --- | --- |
| **Title and abstract** | 1 | (*a*) Indicate the study’s design with a commonly used term in the title or the abstract | 2 | We used a quasi-experimental, matched cohort design using population-based administrative claims databases. |
|  |  | (*b*) Provide in the abstract an informative and balanced summary of what was done and what was found | 2 | Please see “Methods and Findings” section of the abstract |
| Introduction | | | |  |
| Background/rationale | 2 | Explain the scientific background and rationale for the investigation being reported | 4-5 | …recent evidence shows continued high-risk benzodiazepine prescribing to community-dwelling residents Given that the majority of benzodiazepine prescriptions are written by primary care physicians, strategies aimed at improving benzodiazepine prescribing should focus on this group of prescribers. |
| Objectives | 3 | State specific objectives, including any prespecified hypotheses | 5 | Academic detailing (AD) is an educational outreach strategy which has been shown to improve prescribing in primary care…In Ontario, Canada, the Ministry of Health funds an AD initiative in primary care, which focused on benzodiazepine use in older adults from June 2019 to February 2020. The present study is an opportunistic, pragmatic evaluation of this initiative |
| Methods | | | |  |
| Study design | 4 | Present key elements of study design early in the paper | 5 | We conducted a quasi-experimental analysis of benzodiazepine prescribing patterns among family physicians practicing in Ontario. This study used population-based administrative data. Monthly data on benzodiazepine prescribing were collected over a period of 30 months: 12 months pre-and 18 months post-intervention. |
| Setting | 5 | Describe the setting, locations, and relevant dates, including periods of recruitment, exposure, follow-up, and data collection | 5, 7 | See “Design and Setting” (pg 5) and “Population and Exposure” (pg 7) sections for relevant text |
| Participants | 6 | (*a*) *Cohort study*—Give the eligibility criteria, and the sources and methods of selection of participants. Describe methods of follow-up  *Case-control study*—Give the eligibility criteria, and the sources and methods of case ascertainment and control selection. Give the rationale for the choice of cases and controls  *Cross-sectional study*—Give the eligibility criteria, and the sources and methods of selection of participants |  |  |
|  |  | (*b*) *Cohort study*—For matched studies, give matching criteria and number of exposed and unexposed  *Case-control study*—For matched studies, give matching criteria and the number of controls per case | 7 | The intervention (AD) group consisted of family physicians actively practicing in the province of Ontario who voluntarily signed up and received at least one AD visit on benzodiazepine use in older adults between June 2019 and February 2020. Physicians were excluded if they had inactive billing or billing of less than 100 unique patient visits in the calendar year prior to the index date. Index date for physician in the AD group was the date of their first AD visit on benzodiazepine prescribing.  Physicians in the matched control group were included if they never received an AD visit on any topic. We matched each physicians in the AD group to four physicians in the matched group on the following characteristics: index year, region (first letter of postal code), rate of benzodiazepine and opioid agonist therapy (OAT) prescribing in three months prior to index date (yes/no), sex (male/female), active ER status in three months prior to index date (yes/no), detailer is a Family Health Team member (yes/no) and number of years in practice at index (± 5). |
| Variables | 7 | Clearly define all outcomes, exposures, predictors, potential confounders, and effect modifiers. Give diagnostic criteria, if applicable | 8 | The primary outcome was mean total benzodiazepine prescriptions at the level of the physician. This was measured as the total number of benzodiazepine prescriptions written by the physician and dispensed through the Narcotics Monitoring System.  Secondary outcomes included long-term prescriptions, high-risk prescriptions, new start prescriptions, and benzodiazepine-related patient harms. Long-term benzodiazepine prescriptions were defined as 3 continuous prescriptions in a 100-day window. High-risk prescriptions were defined as overlapping benzodiazepine and opioid prescriptions that put the patient at high risk for adverse outcomes, excluding patients with cancer or on palliative care. New start prescriptions were defined as a benzodiazepine prescription to a patient who has not had a claim for this medication in the past year. Finally, benzodiazepine-related harms were measured as the number of benzodiazepine-related hospitalizations, ED visits, falls, or deaths. Secondary outcomes were operationalized as rate (per 100) of patients per month with the occurrence, with the denominator being the number of unique patients with a benzodiazepine prescription in the same month. |
| Data sources/ measurement | 8* | For each variable of interest, give sources of data and details of methods of assessment (measurement). Describe comparability of assessment methods if there is more than one group | 5-6 | Outcomes were assessed using the following population-based administrative claims databases linked through unique identifiers available at ICES: (1) the Ontario Health Insurance Plan (OHIP) database to identify patients and their primary care providers; (2) the Ontario Drug Benefit (ODB) database to assess monthly prescriptions; (3) the Narcotics Monitoring System (NMS) database, which captures all opioid prescriptions dispensed in retail pharmacies across Ontario; (4) the Canadian Institute for Health Information (CIHI) database, which covers all inpatient hospitalizations and emergency department visits; (5) the Discharge Abstracts Database (DAD), National Ambulatory Care Reporting System (NACRS) databases to assign applicable diagnoses; (6) the Registered Persons Database (RPDB), which provides patient demographic information; (7) the ICES Physician Database (IPDB), which provides physician-specific details; and (8) the Drug Identification Number (DIN) database to identify the list of drugs from ODB formularies with DINs, generic and trade names, and strengths. Outcome data was linked with the list of CPSO numbers of the physicians who received and AD visit on this topic, and the date on which the AD visit occurred. |
| Bias | 9 | Describe any efforts to address potential sources of bias | 9 | To account for within-physician dependence of observations, models were fitted using generalized estimating equations and an autoregressive correlation structure. |
| Study size | 10 | Explain how the study size was arrived at | 8 | All family physicians who received an AD visit on benzodiazepine prescribing between June 2019 and February 2020 and met the study inclusion criteria, together with their matched control providers, were included in the analysis. |

Continued on next page

| Quantitative variables | 11 | Explain how quantitative variables were handled in the analyses. If applicable, describe which groupings were chosen and why | 8 | Descriptive statistics were calculated for all physicians and all patients who had a billing code from them during the three months prior to index. Continuous variables with a normal distribution were described using means and standard deviations, and categorical variables were summarized using frequencies and proportions. Analyses were repeated-measures with an intention-to-treat. The unit of analysis was the physician. |
| --- | --- | --- | --- | --- |
| Statistical methods | 12 | (*a*) Describe all statistical methods, including those used to control for confounding | 9 | Analyses of outcomes were planned *a priori* to be performed on the full sample of physicians based on all patient billings during each month of follow-up, as well as on two subgroups: physicians identified as being in the top 25% of benzodiazepine prescribing in the six months prior to index, and patients aged 65 and older.  Outcomes were collected over 12 months pre-and 18 months post-index. To accommodate secular trends, outcomes for the control physicians were collected over the same duration relative to the index date as for their matched exposed physicians. To yield population-averaged estimates of effect, the primary and secondary outcomes were analyzed using segmented negative binomial regression. To account for within-physician dependence of observations, models were fitted using generalized estimating equations and an autoregressive correlation structure. We used the natural log of the number of rostered patients as an offset.  Intervention effects are expressed as monthly percent change in slope together with robust 95% confidence intervals. All analyses were completed using SAS version 9.4 (SAS Institute) at ICES in Toronto, Ontario. Statistical significance was set at *p* < .05, and all tests were 2-tailed. |
|  |  | (*b*) Describe any methods used to examine subgroups and interactions | 9 | Analyses of outcomes were planned *a priori* to be performed on the full sample of physicians based on all patient billings during each month of follow-up, as well as on two subgroups: physicians identified as being in the top 25% of benzodiazepine prescribing in the six months prior to index, and patients aged 65 and older. |
|  |  | (*c*) Explain how missing data were addressed | NA | NA |
|  |  | (*d*) *Cohort study*—If applicable, explain how loss to follow-up was addressed  *Case-control study*—If applicable, explain how matching of cases and controls was addressed  *Cross-sectional study*—If applicable, describe analytical methods taking account of sampling strategy | 7 | We matched each physicians in the AD group to four physicians in the matched group on the following characteristics: index year, region (first letter of postal code), rate of benzodiazepine and opioid agonist therapy (OAT) prescribing in three months prior to index date (yes/no), sex (male/female), active ER status in three months prior to index date (yes/no), detailer is a Family Health Team member (yes/no) and number of years in practice at index (± 5). Quality checking of the resulting matches included comparing distributions of baseline variables for the two groups using standardized differences. Index date was randomly assigned to matched control physicians based on the frequency distribution of index years among AD physicians. |
|  |  | (*e*) Describe any sensitivity analyses | 9 | An additional sensitivity analysis was performed for physicians in the top 25% of benzodiazepine prescribing in the one month prior to index. |
| Results | | | | |
| Participants | 13* | (a) Report numbers of individuals at each stage of study—eg numbers potentially eligible, examined for eligibility, confirmed eligible, included in the study, completing follow-up, and analysed | 9 | A total of 1337 primary care physicians were included in the study, with 273 (20%) in the AD group and 1064 (80%) as matched controls. |
|  |  | (b) Give reasons for non-participation at each stage | NA | NA |
|  |  | (c) Consider use of a flow diagram | NA | NA |
| Descriptive data | 14* | (a) Give characteristics of study participants (eg demographic, clinical, social) and information on exposures and potential confounders | 9-10 | As seen in Table 1, physicians in the AD and matched control groups were well-balanced, although physicians in the AD group had a slightly smaller average roster size compared to the matched controls (1632.47 ± 920.05 vs. 1770.59 ± 1339.24). Details on types of benzodiazepines prescribed can be found in Table S1 in the appendix. |
|  |  | (b) Indicate number of participants with missing data for each variable of interest | NA | NA |
|  |  | (c) *Cohort study*—Summarise follow-up time (eg, average and total amount) | NA | NA |
| Outcome data | 15* | *Cohort study*—Report numbers of outcome events or summary measures over time | 12-15 | Over a 30-month period (12 months pre-intervention to 18 months post-intervention), the mean total number of benzodiazepine prescriptions per month decreased among physicians in both the AD group (32.47 to 30.61) and matched control group (31.95 to 26.28, see Fig 1). The decrease in total prescriptions for both groups began in the pre-intervention period, with matched controls showing a small but significant monthly percent change (-0.51, 95% CI = -0.92 to -0.10, *p* = .01), but not the AD group (-0.49, 95%CI = -1.21 to 0.23, *p* = .18). During the post-intervention period, neither group had a significant monthly percent change from pre-intervention and there were no significant differences between groups (see Table 3). |
|  |  | *Case-control study—*Report numbers in each exposure category, or summary measures of exposure |  |  |
|  |  | *Cross-sectional study—*Report numbers of outcome events or summary measures |  |  |
| Main results | 16 | (*a*) Give unadjusted estimates and, if applicable, confounder-adjusted estimates and their precision (eg, 95% confidence interval). Make clear which confounders were adjusted for and why they were included | 12-15 | Over a 30-month period (12 months pre-intervention to 18 months post-intervention), the mean total number of benzodiazepine prescriptions per month decreased among physicians in both the AD group (32.47 to 30.61) and matched control group (31.95 to 26.28, see Fig 1). The decrease in total prescriptions for both groups began in the pre-intervention period, with matched controls showing a small but significant monthly percent change (-0.51, 95% CI = -0.92 to -0.10, *p* = .01), but not the AD group (-0.49, 95%CI = -1.21 to 0.23, *p* = .18). During the post-intervention period, neither group had a significant monthly percent change from pre-intervention and there were no significant differences between groups (see Table 3). |
|  |  | (*b*) Report category boundaries when continuous variables were categorized | NA | NA |
|  |  | (*c*) If relevant, consider translating estimates of relative risk into absolute risk for a meaningful time period | NA | NA |

Continued on next page

| Other analyses | 17 | Report other analyses done—eg analyses of subgroups and interactions, and sensitivity analyses | 14-15 | Over a 30-month period, the total number of prescriptions decreased among high prescribing physicians in both the AD group (72.76 to 61.76) and matched controls (37.24 to 29.22, see Fig 3). The percent change in slope from pre-intervention to 18-months post intervention was greater for the AD group compared to the matched controls, however this difference was not significant (see Table 3). Similar trends were seen in the sensitivity analysis (see S13 Fig). |
| --- | --- | --- | --- | --- |
| Discussion | | | | |
| Key results | 18 | Summarise key results with reference to study objectives | 15-16 | In this quasi-experimental study, analyses indicate no significant overall effect of a real-world AD initiative on benzodiazepine prescribing. Overall, total monthly prescriptions and new prescriptions showed small decreases in the post-intervention period, while long-term prescriptions, high-risk prescriptions and benzodiazepine-related patient harms increased post-intervention. Interestingly, higher-prescribing physicians receiving AD showed a greater but non-significant reduction in their benzodiazepine prescribing compared to their matched controls. This suggests a potential opportunity to optimize the effects of an AD intervention by focusing the offering to those physicians with greatest room for improvement. |
| Limitations | 19 | Discuss limitations of the study, taking into account sources of potential bias or imprecision. Discuss both direction and magnitude of any potential bias | 17-18 | The intervention group of our study was limited to physicians who volunteered to receive the AD intervention, which may have introduced a self-selection bias of physicians most open to changing their prescribing… Given the retrospective, quasi-experimental design of this study, the data were not collected specifically for the study, but rather obtained through prescription databases. Patients not captured within the administrative databases (i.e. non-valid OHIP and prescriptions from non-primary care physicians) were excluded. It is possible that other effects may have been achieved in other safe-prescribing practices not easily measured through administrative data. |
| Interpretation | 20 | Give a cautious overall interpretation of results considering objectives, limitations, multiplicity of analyses, results from similar studies, and other relevant evidence | 18-19 | In summary, this quasi-experimental study evaluating a real-world intervention aiming to improve benzodiazepine prescribing in primary care found that a single AD visit was not associated with additional changes at a population level beyond those seen in secular trends. The voluntary nature of the intervention meant that some who engaged may have had little room for improvement. Those with greatest room for improvement in their prescribing behaviour seemed to benefit most. Despite these findings, past research has supported the use of AD as a means to improve appropriate prescribing of benzodiazepines. In the future, the effectiveness of AD may be enhanced by using a more targeted and data-driven approach to offering visits to physicians with room for improvement to their prescribing, as well as offering multiple or longer sessions. |
| Generalisability | 21 | Discuss the generalisability (external validity) of the study results | 18-19 | Despite these findings, past research has supported the use of AD as a means to improve appropriate prescribing of benzodiazepines. In the future, the effectiveness of AD may be enhanced by using a more targeted and data-driven approach to offering visits to physicians with room for improvement to their prescribing, as well as offering multiple or longer sessions. |
| Other information | |  | | |
| Funding | 22 | Give the source of funding and the role of the funders for the present study and, if applicable, for the original study on which the present article is based | 19 | Funding for this project was provided by the Centre for Effective Practice, which is funded by the Ontario Ministry of Health. Parts of this material are based on data and information compiled and provided by CIHI, Ontario Health (OH) and the Ontario Ministry of Health. The analyses, conclusions, opinions, and statements expressed herein are solely those of the authors and do not reflect those of the funding or data sources; no endorsement is intended or should be inferred. We thank IQVIA Solutions Canada Inc. for use of their Drug Information File. |

*Give information separately for cases and controls in case-control studies and, if applicable, for exposed and unexposed groups in cohort and cross-sectional studies.

**Note:** An Explanation and Elaboration article discusses each checklist item and gives methodological background and published examples of transparent reporting. The STROBE checklist is best used in conjunction with this article (freely available on the Web sites of PLoS Medicine at http://www.plosmedicine.org/, Annals of Internal Medicine at http://www.annals.org/, and Epidemiology at http://www.epidem.com/). Information on the STROBE Initiative is available at www.strobe-statement.org.
