# Supplementary figures and images for: Effects of an academic detailing service on benzodiazepine prescribing patterns in primary care
Source: PLoS One. 2023 Jul 27;18(7):e0289147. doi: 10.1371/journal.pone.0289147 (PMC10374092; doi:10.1371/journal.pone.0289147)

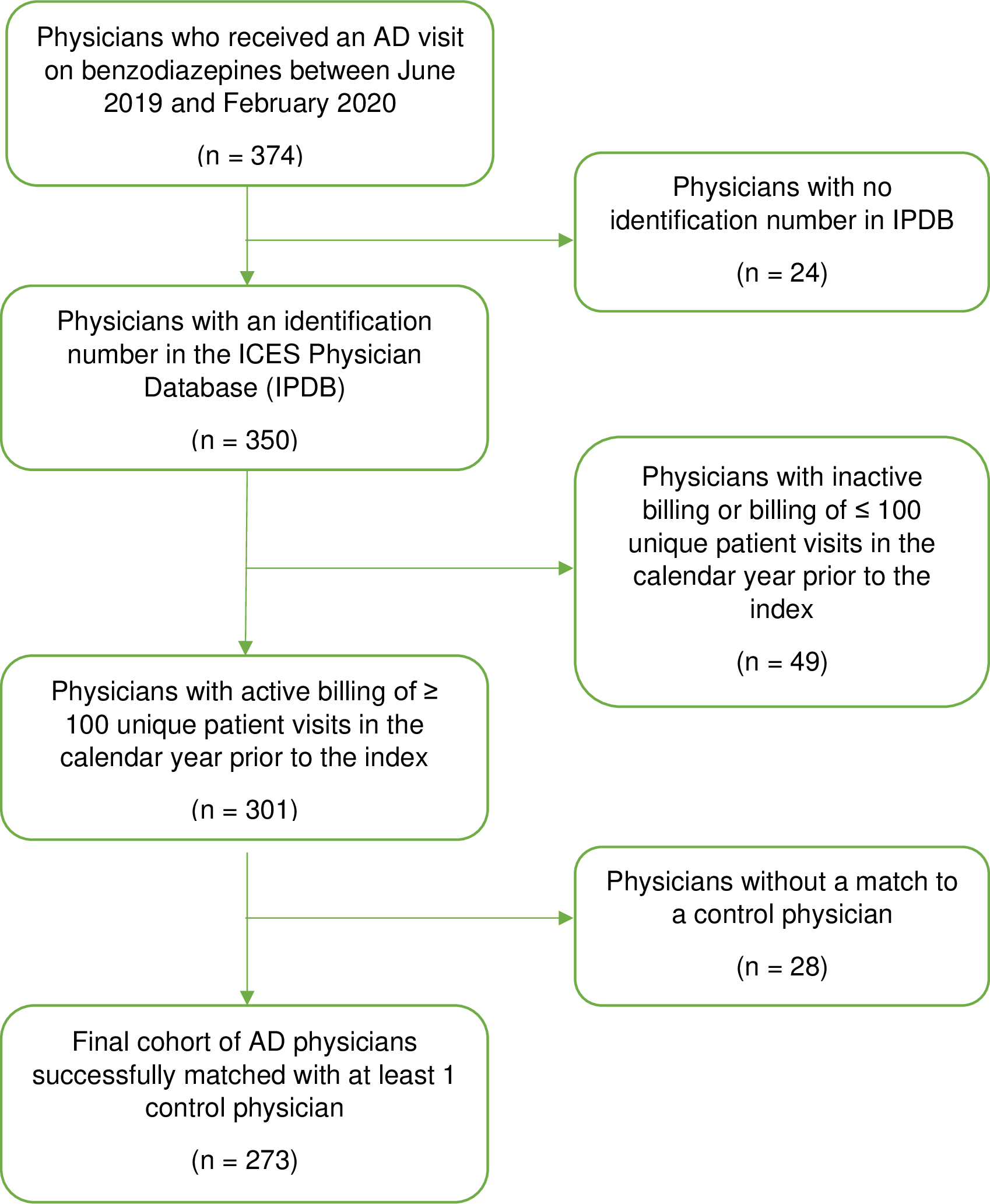

Supplement: S1 Fig — (TIF) [file pone.0289147.s002.tif]

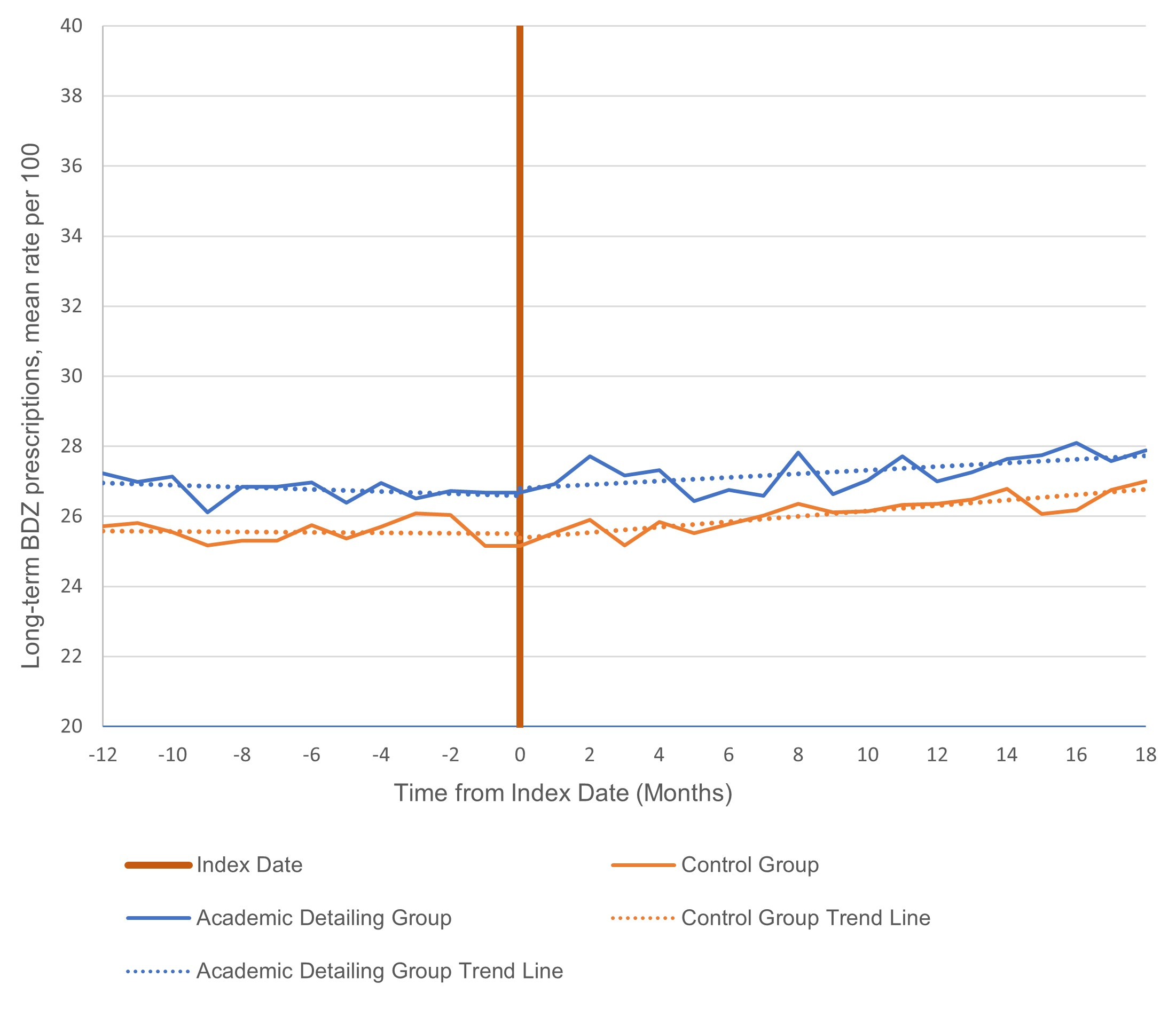

Supplement: S2 Fig — (TIF) [file pone.0289147.s003.tif]

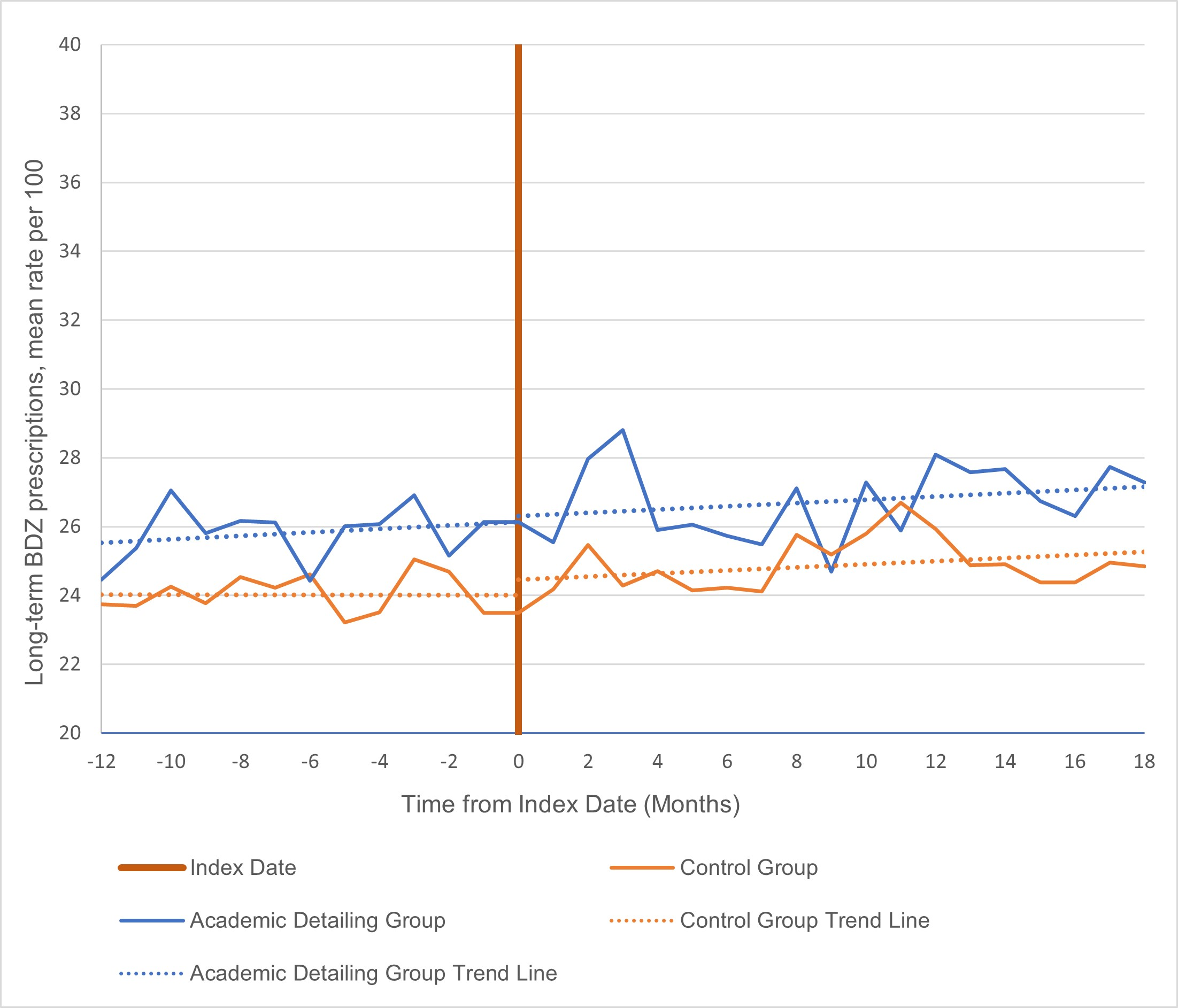

Supplement: S3 Fig — (TIF) [file pone.0289147.s004.tif]

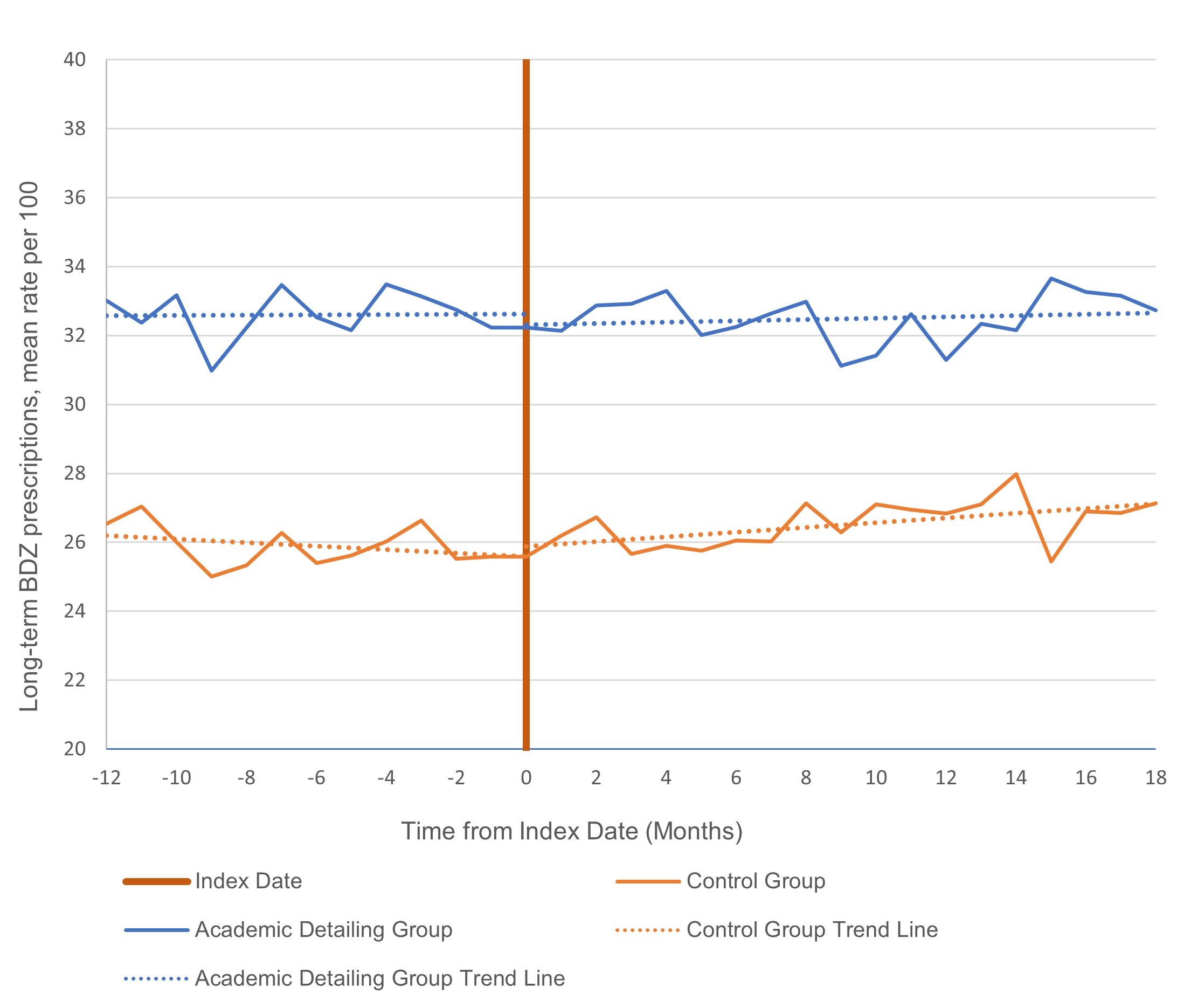

Supplement: S4 Fig — (TIF) [file pone.0289147.s005.tif]

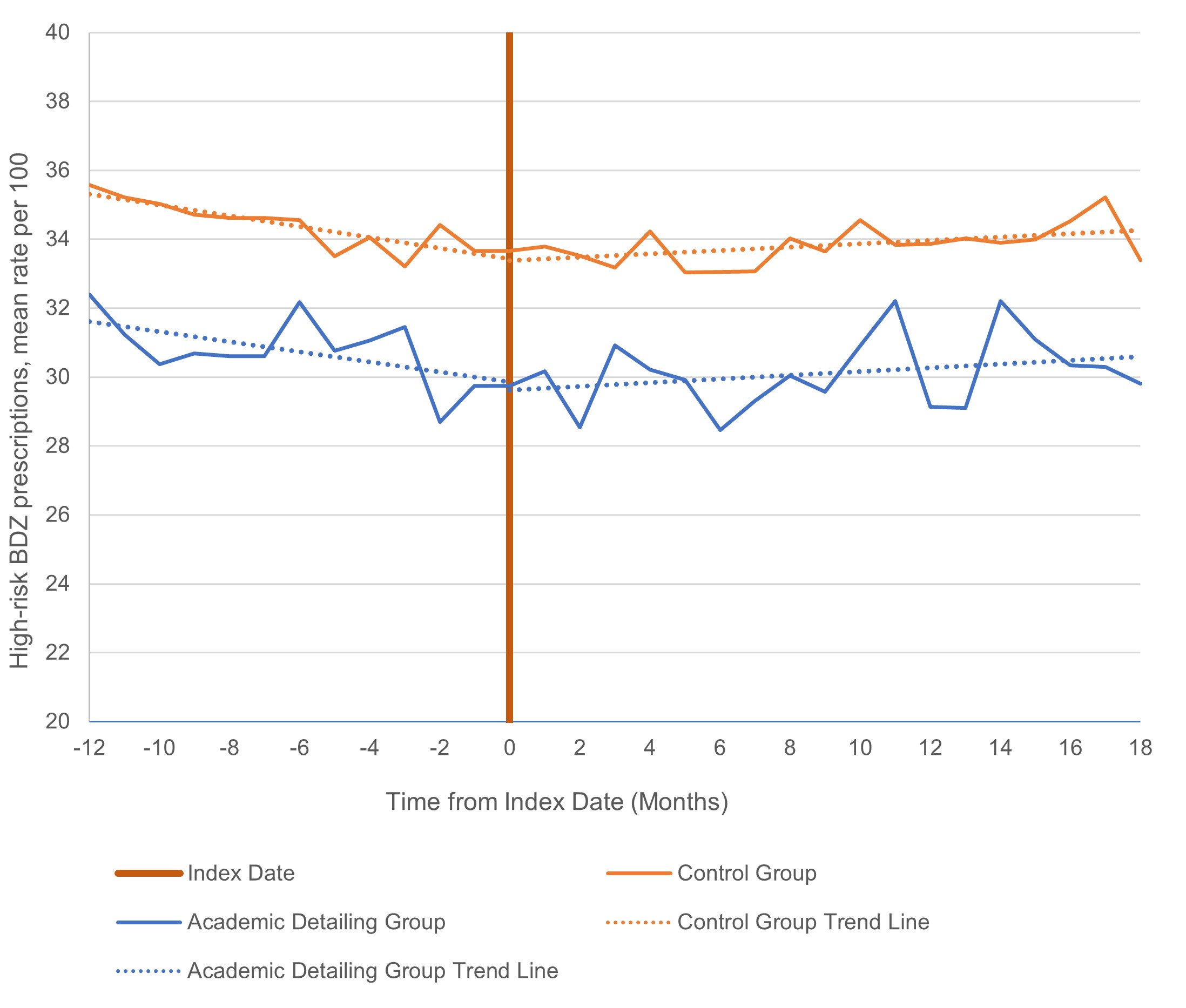

Supplement: S5 Fig — (TIF) [file pone.0289147.s006.tif]

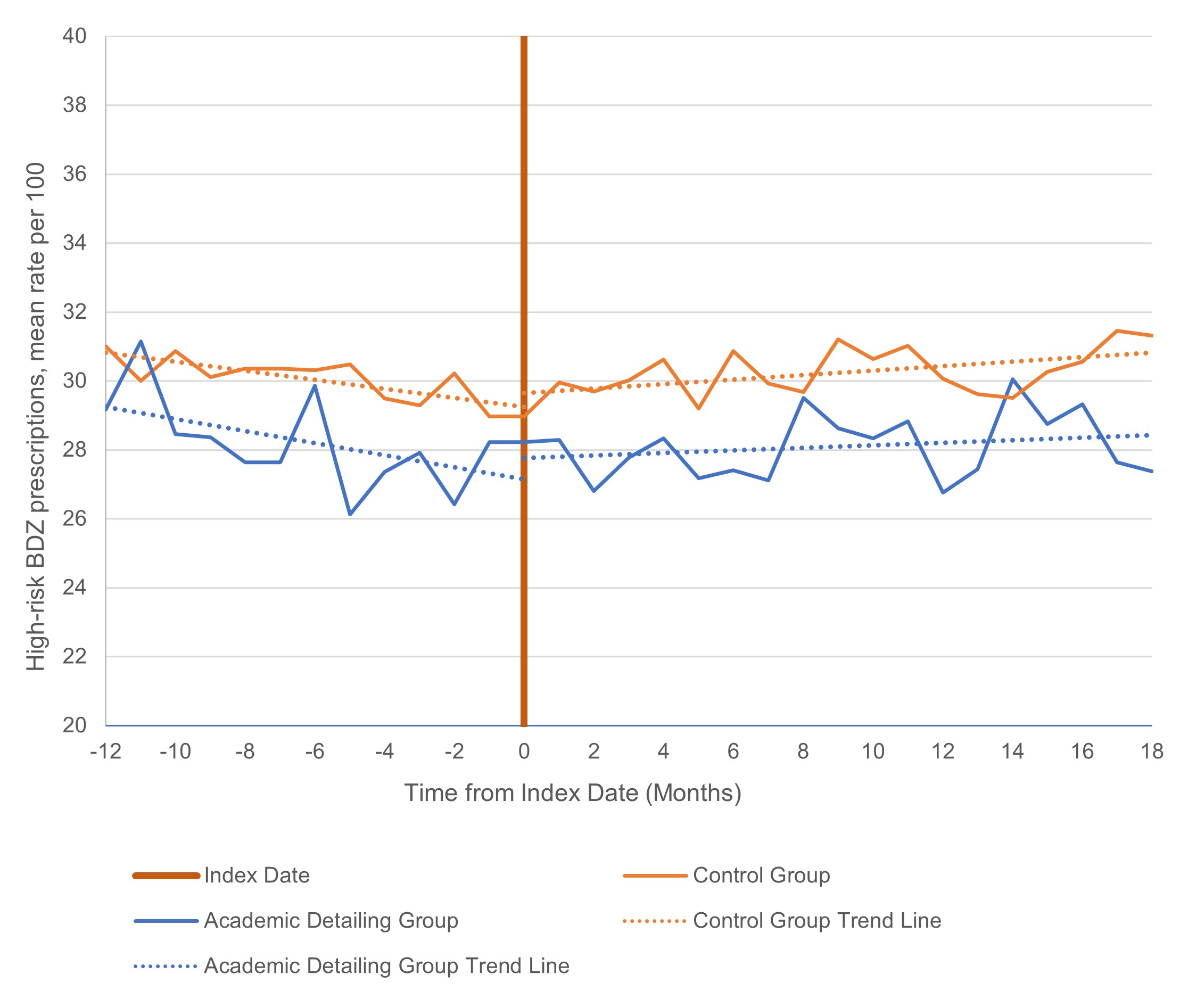

Supplement: S6 Fig — (TIF) [file pone.0289147.s007.tif]

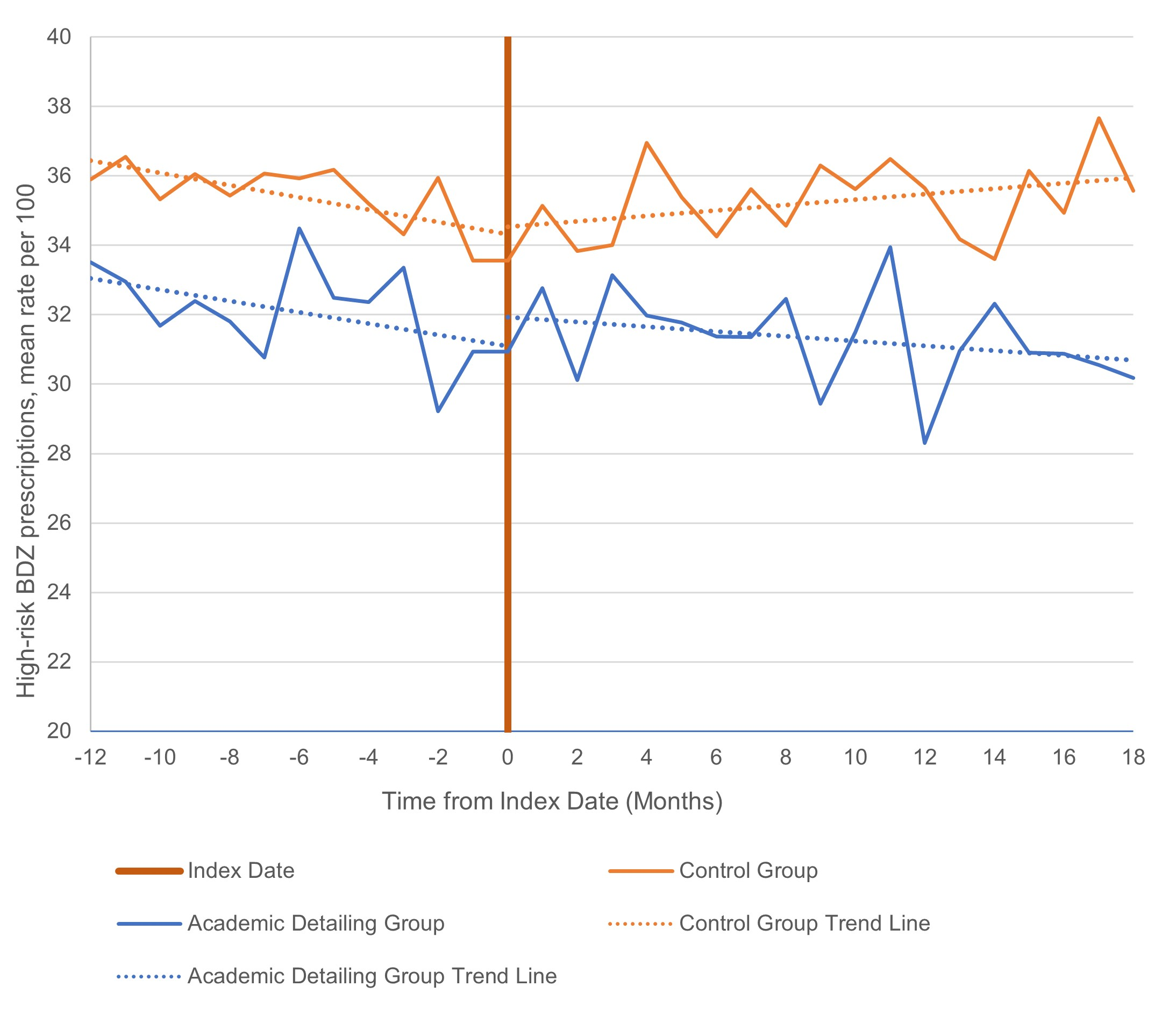

Supplement: S7 Fig — (TIF) [file pone.0289147.s008.tif]

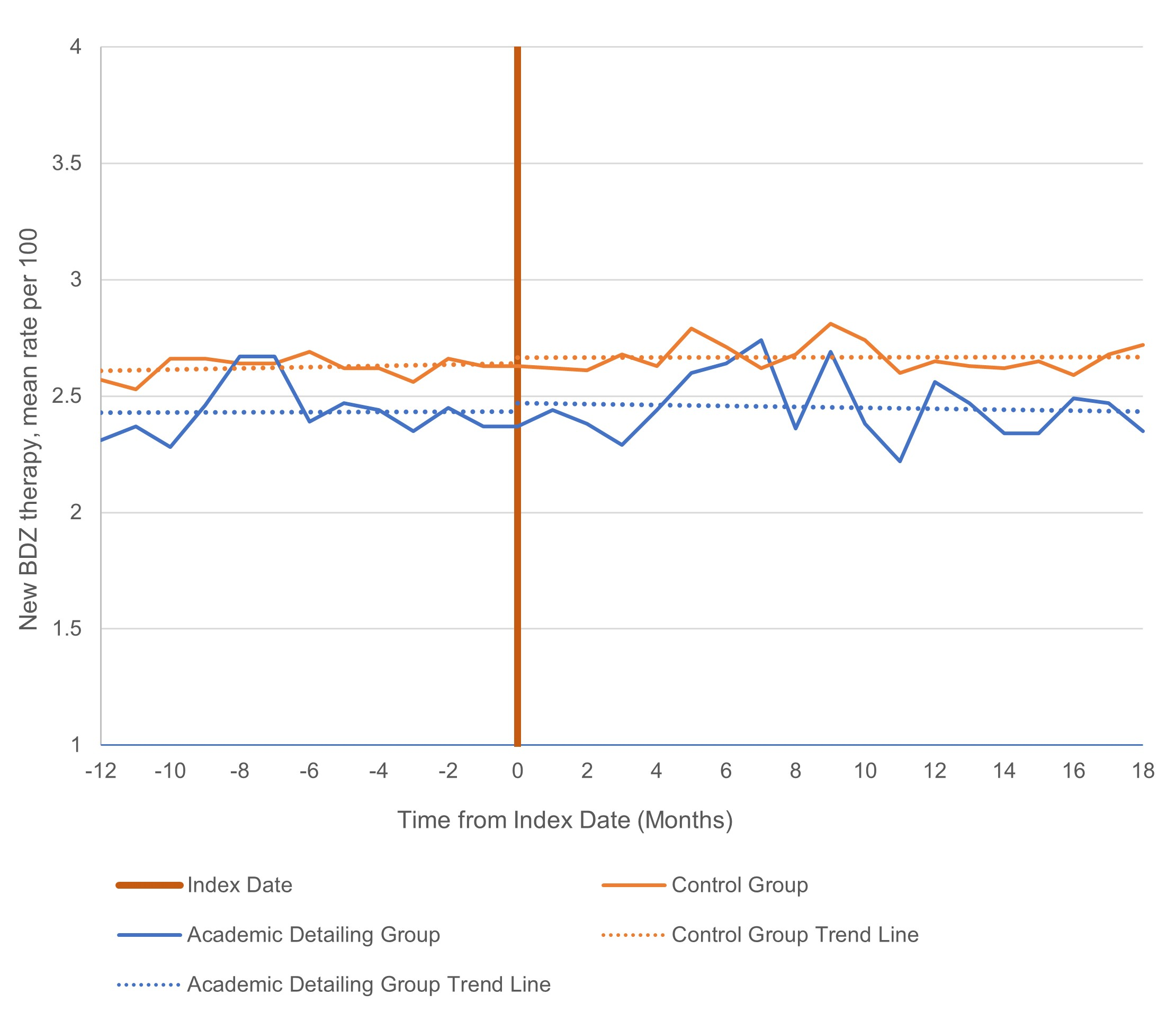

Supplement: S8 Fig — (TIF) [file pone.0289147.s009.tif]

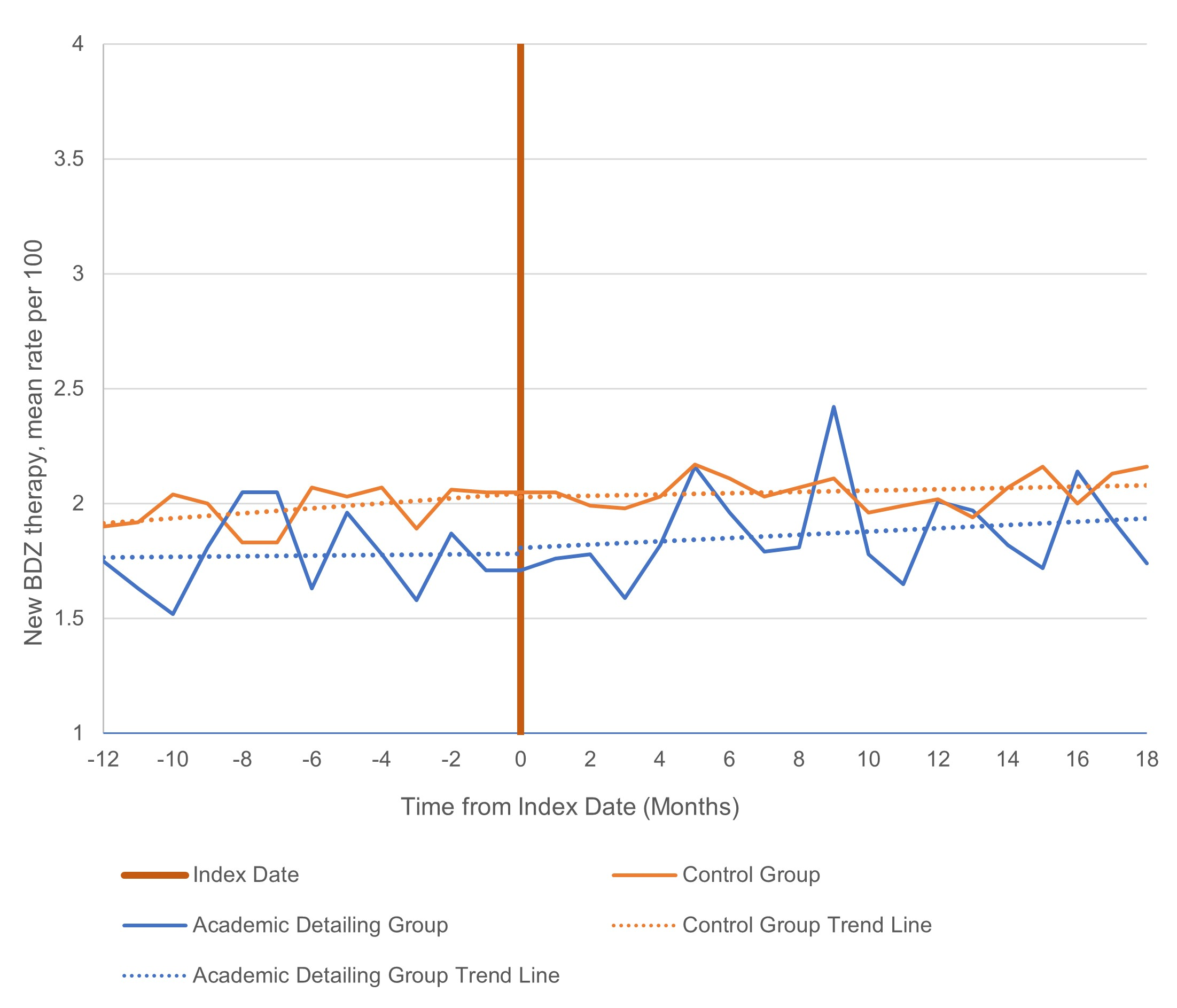

Supplement: S9 Fig — (TIF) [file pone.0289147.s010.tif]

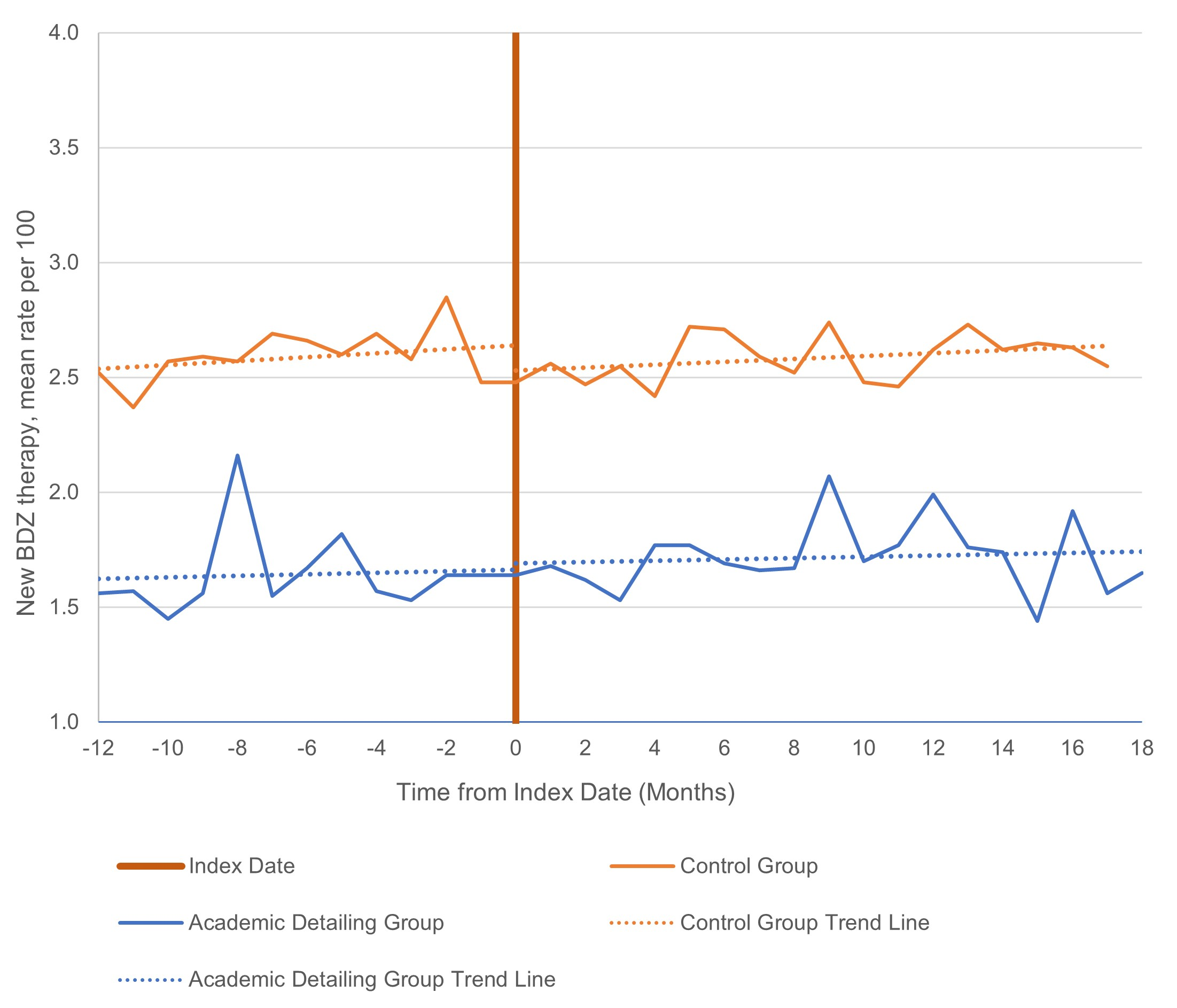

Supplement: S10 Fig — (TIF) [file pone.0289147.s011.tif]

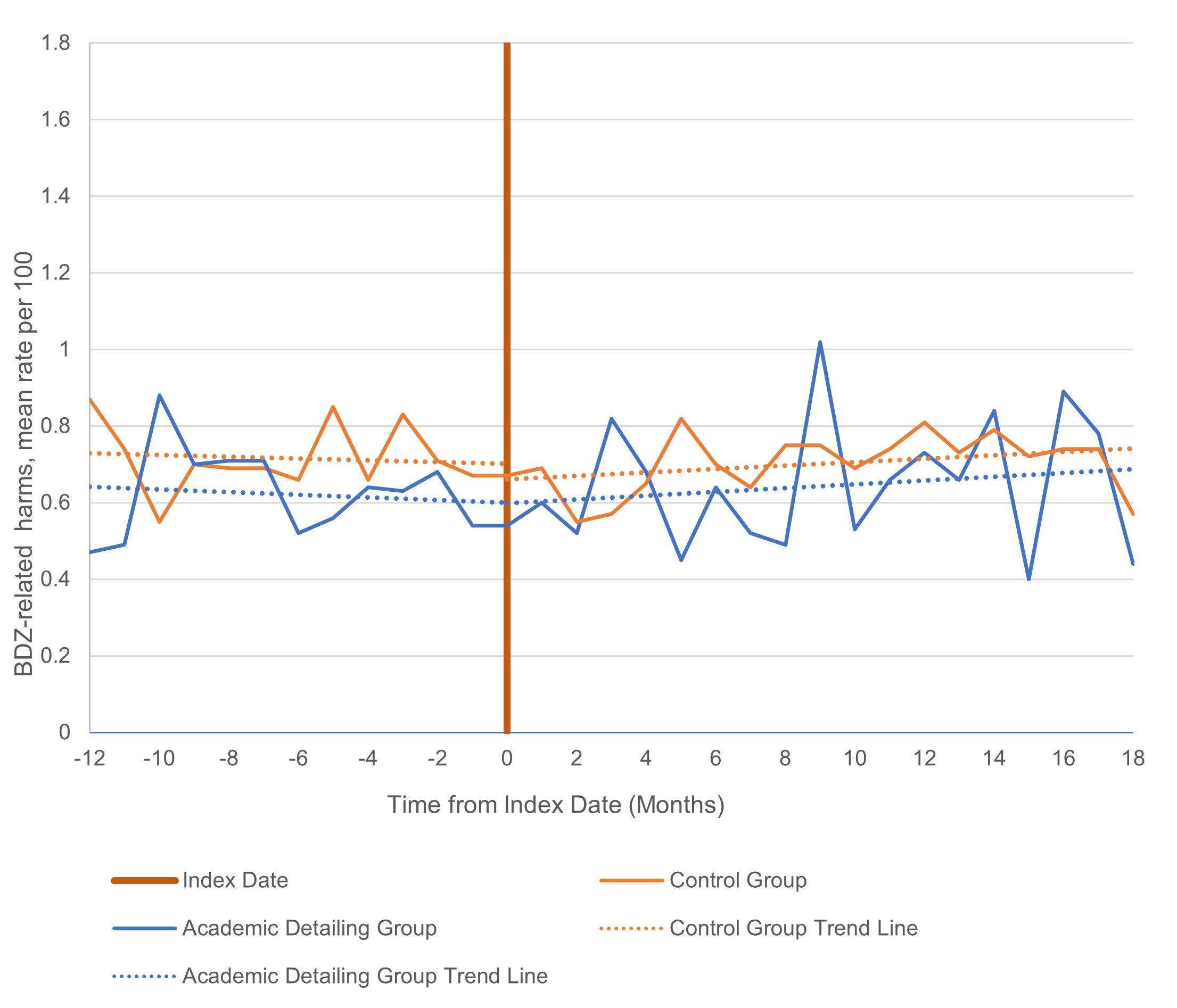

Supplement: S11 Fig — (TIF) [file pone.0289147.s012.tif]

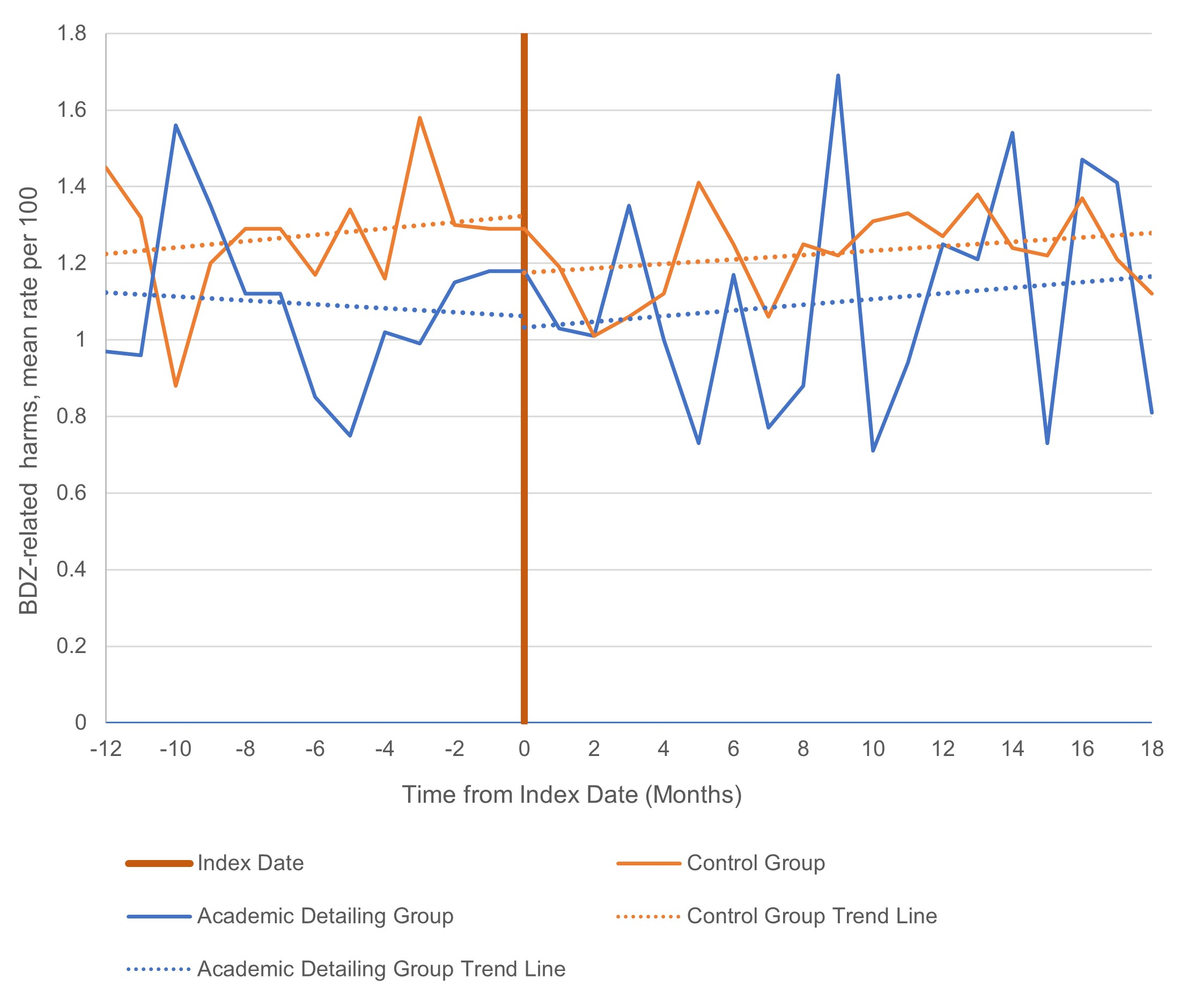

Supplement: S12 Fig — (TIF) [file pone.0289147.s013.tif]

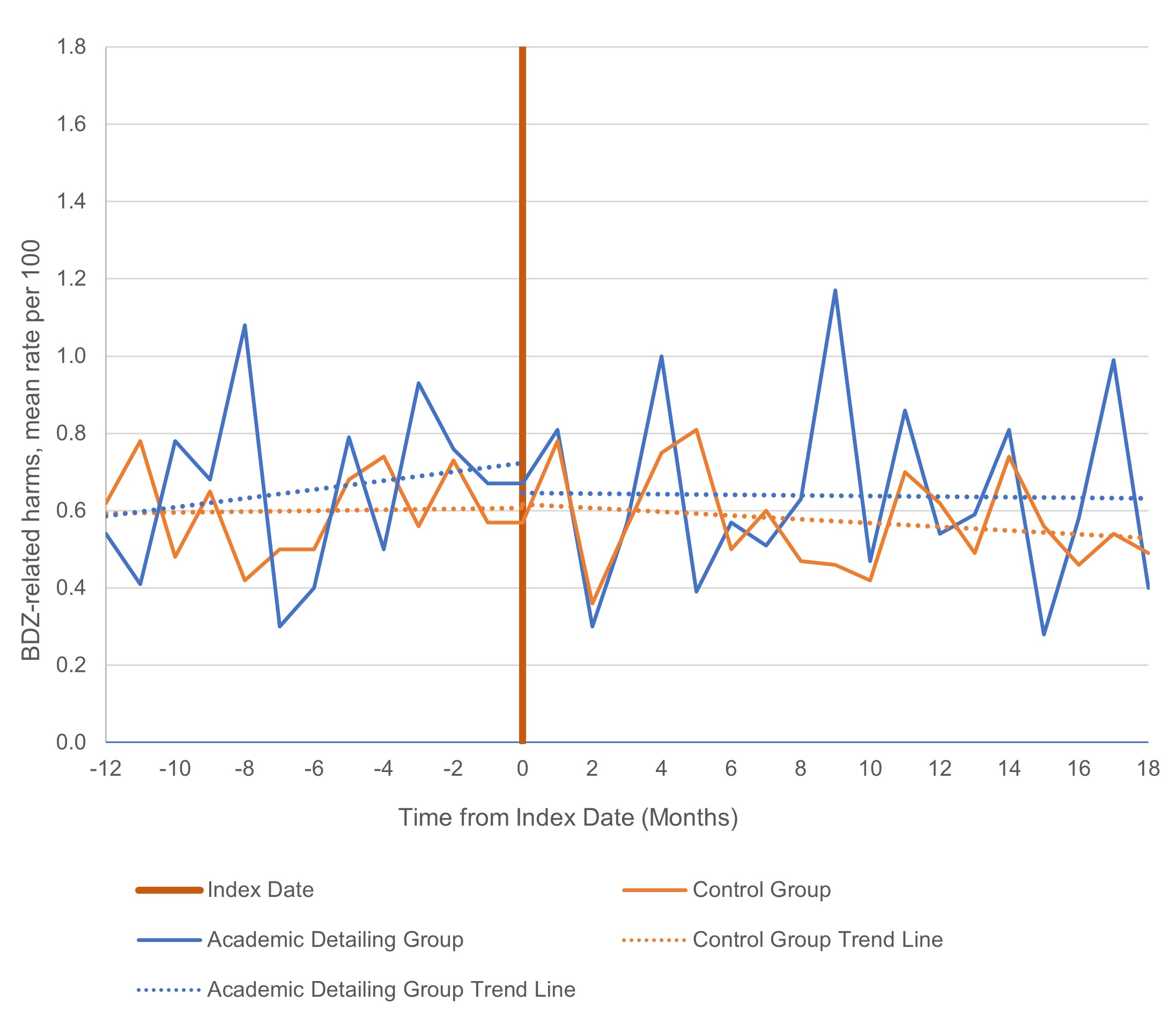

Supplement: S13 Fig — (TIF) [file pone.0289147.s014.tif]

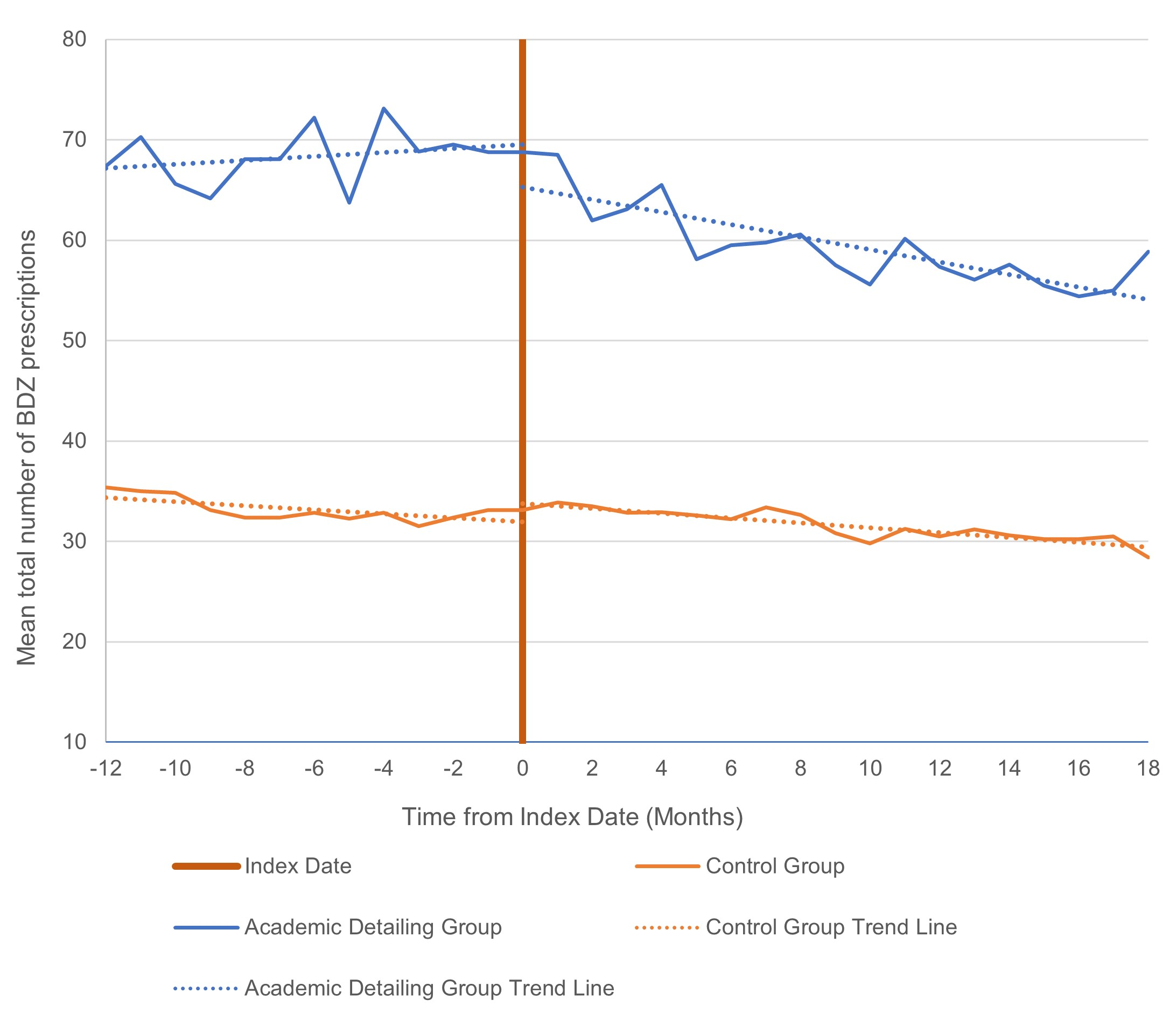

Supplement: S14 Fig — (TIF) [file pone.0289147.s015.tif]

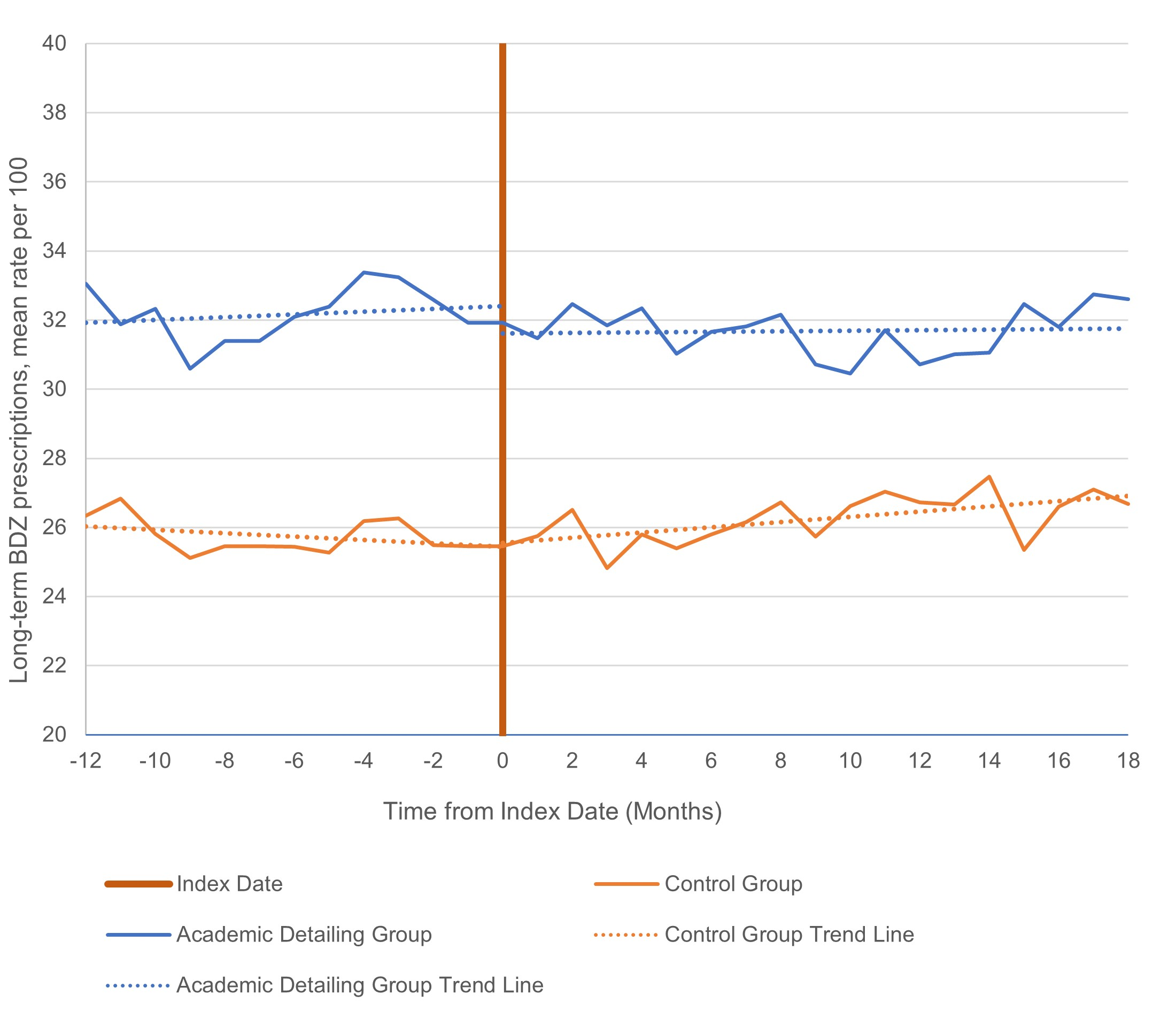

Supplement: S15 Fig — (TIF) [file pone.0289147.s016.tif]

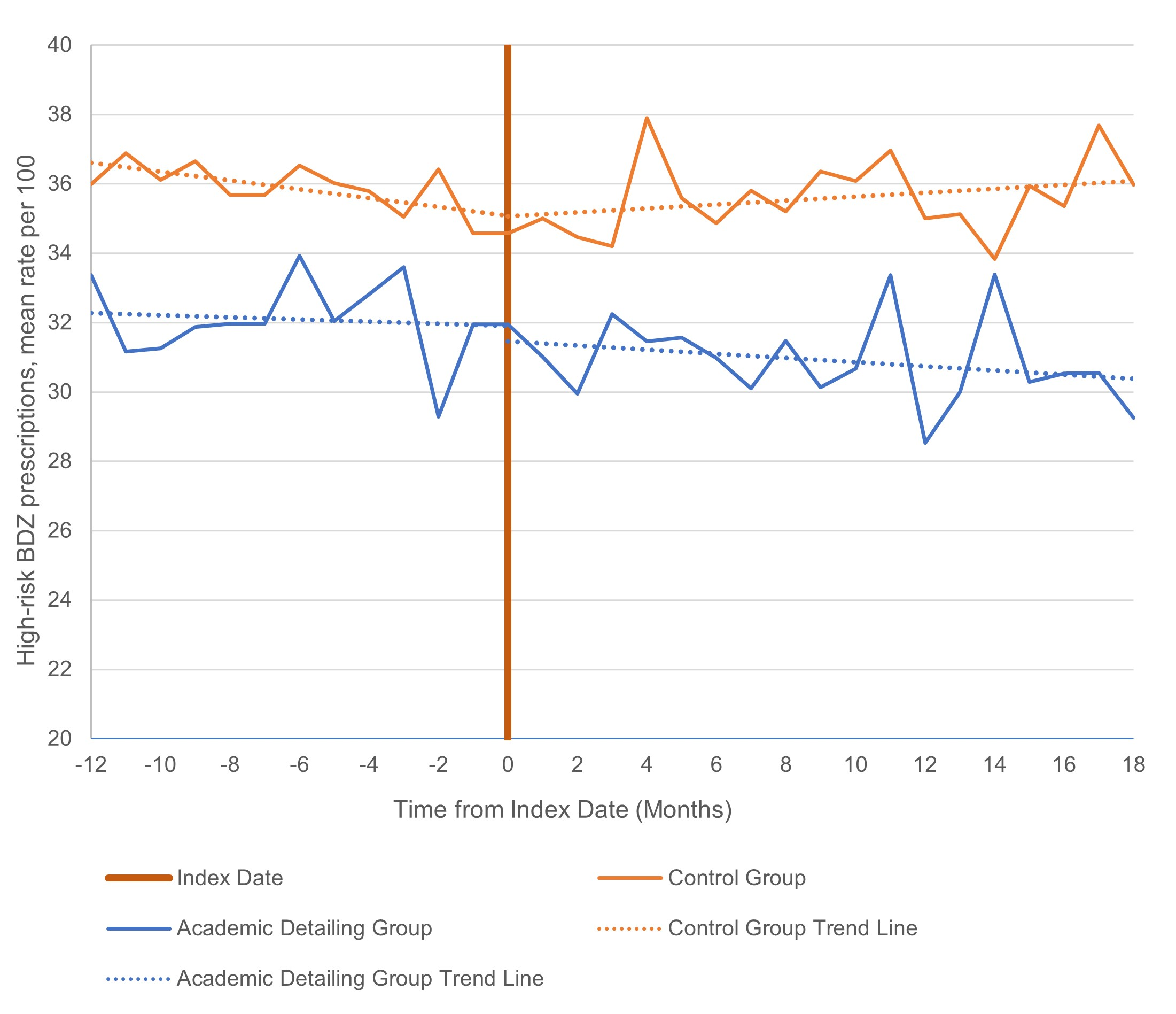

Supplement: S16 Fig — (TIF) [file pone.0289147.s017.tif]

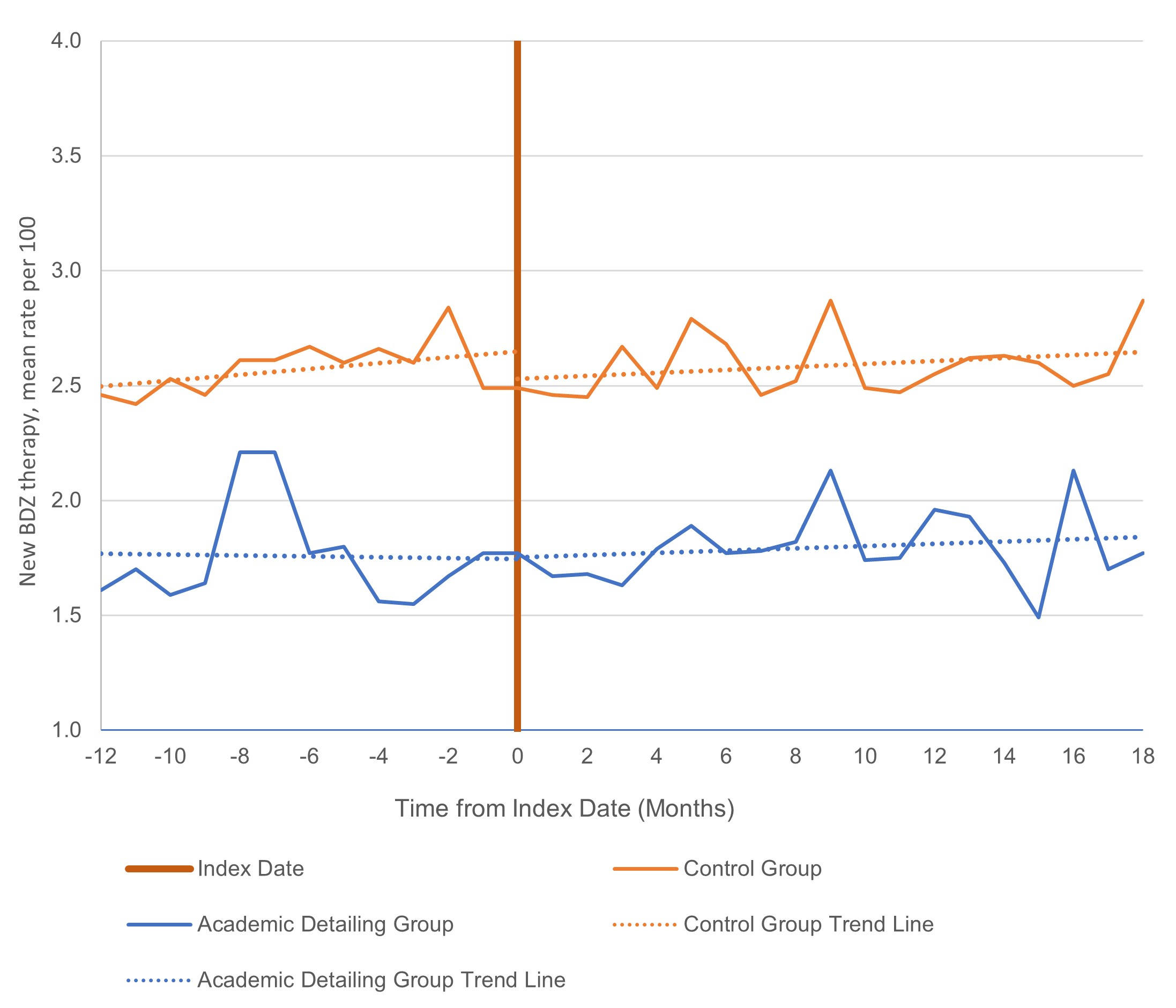

Supplement: S17 Fig — (TIF) [file pone.0289147.s018.tif]

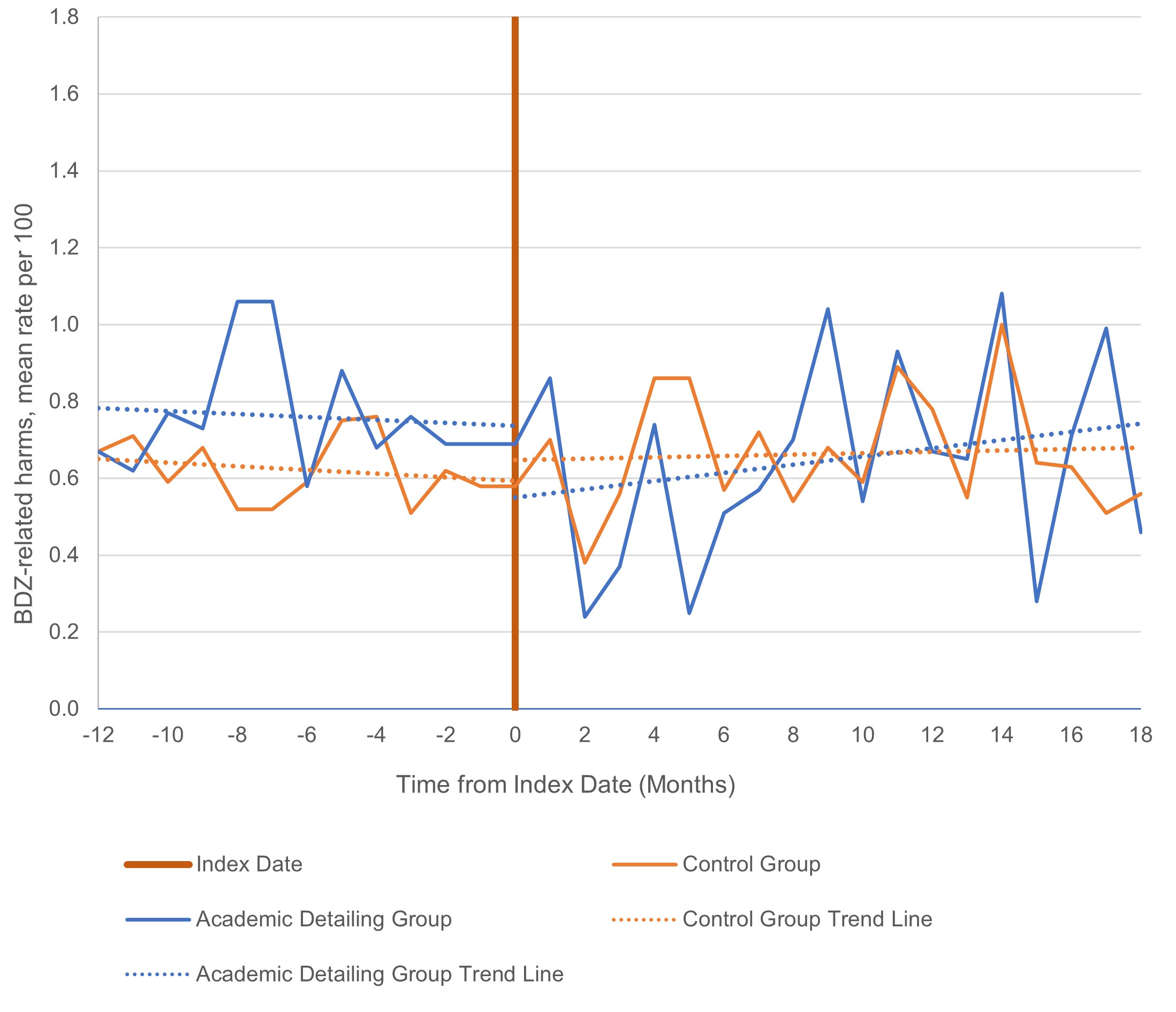

Supplement: S18 Fig — (TIF) [file pone.0289147.s019.tif]
