# Supplementary material for: Effects of an academic detailing service on benzodiazepine prescribing patterns in primary care
Source: PLoS One. 2023 Jul 27;18(7):e0289147. doi: 10.1371/journal.pone.0289147 (PMC10374092; doi:10.1371/journal.pone.0289147)
Supplement: S1 Table — (PDF) [file pone.0289147.s020.pdf]

S1 Table. Types of Benzodiazepines Received by Patients

Benzodiazepine Type	No(%)		
	**Academic Detailing n=14244**	**Control n=55088**	**Standardized Difference**
Short-acting			
Midazolam (Versed)	214 (1.6)	1069 (2.0)	0.03
Triazolam (Halcion)	72 (0.5)	206 (0.4)	0.02
Immediate-acting			
Alprazolam (Xanax)	490 (3.6)	2429 (4.5)	0.05
Bromazepam (Lectopam)	83 (0.6)	488 (0.9)	0.04
Clobazam (Frisium)	165 (1.2)	684 (1.3)	0.01
Clonazepam (Rivotril)	3851 (27.9)	14261 (26.7)	0.03
Lorazepam (Ativan)	7923 (57.4)	30062 (56.3)	0.02
Nitrazepam (Mogadon)	95 (0.7)	582 (1.1)	0.04
Oxazepam (Serax)	677 (4.9)	2369 (4.4)	0.02
Temazepam (Restoril)	525 (3.8)	2933 (5.5)	0.08
Long-acting			
Chlordiazepoxide (Librium)	21 (0.2)	93 (0.2)	0.01
Clorazepate (Tranxene)	9 (0.1)	55 (0.1)	0.01
Diazepam (Valium)	652 (4.7)	2533 (4.7)	0.00
Flurazepam (Dalmane)	19 (0.1)	145 (0.3)	0.03
Other	101 (0.7)	580 (1.1)	0.04
New-start benzodiazepine therapy	60 (5.8)	447 (6.4)	0.02
Long-term benzodiazepine therapy	227 (21.9)	1441 (20.5)	0.03
