# Supplementary material for: Effects of an academic detailing service on benzodiazepine prescribing patterns in primary care
Source: PLoS One. 2023 Jul 27;18(7):e0289147. doi: 10.1371/journal.pone.0289147 (PMC10374092; doi:10.1371/journal.pone.0289147)
Supplement: S2 Table — (PDF) [file pone.0289147.s021.pdf]

S2 Table. Types of Benzodiazepines Received by Patients of High Prescribers

	No(%)		
**Benzodiazepine Type**	**Academic Detailing n=6038**	**Control n=14679**	**Standardized Difference**
Short-acting			
Midazolam (Versed)	98 (1.7)	382 (2.7)	0.07
Triazolam (Halcion)	25 (0.4)	61 (0.4)	0.00
Immediate-acting			
Alprazolam (Xanax)	193 (3.3)	630 (4.4)	0.06
Bromazepam (Lectopam)	51 (0.9)	133 (0.9)	0.01
Clobazam (Frisium)	57 (1.0)	175 (1.2)	0.02
Clonazepam (Rivotril)	1699 (28.8)	3718 (26.0)	0.06
Lorazepam (Ativan)	3308 (56.1)	8079 (56.4)	0.01
Nitrazepam (Mogadon)	57 (1.0)	201 (1.4)	0.04
Oxazepam (Serax)	333 (5.6)	613 (4.3)	0.06
Temazepam (Restoril)	302 (5.1)	795 (5.6)	0.02
Long-acting			
Chlordiazepoxide (Librium)	13 (0.2)	27 (0.2)	0.01
Clorazepate (Tranxene)	<=5* (0.1)	18 (0.1)	0.02
Diazepam (Valium)	268 (4.5)	702 (4.9)	0.02
Flurazepam (Dalmane)	9 (0.2)	50 (0.3)	0.04
Other	56 (0.9)	161 (1.1)	0.02
New-start BDZ therapy	39 (6.5)	135 (5.9)	0.02
Long-term BDZ therapy	125 (20.8)	455 (19.9)	0.02

* ICES' *Protection of ICES Data* policy prohibits inclusion of small cells of fewer than five in any report or publication of the results of any ICES project or any research
