# Supplementary material for: Effects of an academic detailing service on benzodiazepine prescribing patterns in primary care
Source: PLoS One. 2023 Jul 27;18(7):e0289147. doi: 10.1371/journal.pone.0289147 (PMC10374092; doi:10.1371/journal.pone.0289147)
Supplement: S3 Table — (PDF) [file pone.0289147.s022.pdf]

S3 Table. Types of Benzodiazepines Received by Patients >65

	No(%)		
**Benzodiazepine Type**	**Academic Detailing n=6215**	**Control n=22715**	**Standardized Difference**
Short-acting			
Midazolam (Versed)	181 (3.0)	855 (3.9)	0.05
Triazolam (Halcion)	31 (0.5)	88 (0.4)	0.02
Immediate-acting			
Alprazolam (Xanax)	235 (3.9)	1,008 (4.6)	0.03
Bromazepam (Lectopam)	61 (1.0)	316 (1.4)	0.04
Clobazam (Frisium)	59 (1.0)	194 (0.9)	0.01
Clonazepam (Rivotril)	1222 (20.2)	4285 (19.4)	0.02
Lorazepam (Ativan)	3527 (58.4)	12600 (57.0)	0.03
Nitrazepam (Mogadon)	50 (0.8)	289 (1.3)	0.05
Oxazepam (Serax)	485 (8.0)	1593 (7.2)	0.03
Temazepam (Restoril)	290 (4.8)	1485 (6.7)	0.08
Long-acting			
Chlordiazepoxide (Librium)	14 (0.2)	41 (0.2)	0.01
Clorazepate (Tranxene)	<=5* (0.1)	38 (0.2)	0.03
Diazepam (Valium)	248 (4.1)	890 (4.0)	0.00
Flurazepam (Dalmane)	13 (0.2)	73 (0.3)	0.02
Other	73 (1.2)	361 (1.6)	0.04
New-start BDZ therapy	32 (7.1)	214 (7.2)	0.00
Long-term BDZ therapy	89 (19.8)	593 (19.9)	0.00

* ICES' *Protection of ICES Data* policy prohibits inclusion of small cells of fewer than five in any report or publication of the results of any ICES project or any research
