# Supplementary material for: Effects of an academic detailing service on benzodiazepine prescribing patterns in primary care
Source: PLoS One. 2023 Jul 27;18(7):e0289147. doi: 10.1371/journal.pone.0289147 (PMC10374092; doi:10.1371/journal.pone.0289147)
Supplement: S4 Table — (PDF) [file pone.0289147.s023.pdf]

S4 Table. Baseline Characteristics of Patients > 65 Years

**Senior Patient (65+) Characteristics (prior 3 months)**	No. (%)		
	**Academic Detailing n=59483**	**Control n=206853**	**Standardized Difference**
Male	25824 (43.4)	89889 (43.5)	0.00
Age (mean ,SE)	75.90 ± 8.11	75.68 ± 7.99	0.03
Income quintile			
1 (lowest)	12300 (20.7)	42966 (20.8)	0.00
2	11846 (19.9)	42639 (20.6)	0.02
3	12148 (20.4)	40832 (19.7)	0.02
4	11844 (19.9)	38852 (18.8)	0.03
5 (highest)	11171 (18.8)	40710 (19.7)	0.02
Cancer	2382 (4.0)	8327 (4.0)	0.00
Palliative status	1147 (1.9)	3930 (1.9)	0.00
History of mental illness	9876 (16.6)	34830 (16.8)	0.01
Psychiatry consultation in year prior	2384 (4.0)	7440 (3.6)	0.02
Any benzodiazepine prescription	6215 (10.4)	22715 (11.0)	0.02
