# Supplementary material for: Effects of an academic detailing service on benzodiazepine prescribing patterns in primary care
Source: PLoS One. 2023 Jul 27;18(7):e0289147. doi: 10.1371/journal.pone.0289147 (PMC10374092; doi:10.1371/journal.pone.0289147)
Supplement: S5 Table. Number of academic detailing sessions attended on any topic — (PDF) [file pone.0289147.s024.pdf]

S5 Table. Number of Academic Detailing Sessions Attended on Any Topic*

	No. (%)	
**Number of sessions (all topics)**	**All Physicians (N = 273)**	**Top Prescribers (N = 67)**
1	29 (11)	<6 (<10)
2	37 (13)	<6 (<10)
3	59 (22)	17 (26)
4	101 (37)	32 (48)
5+	46 (17)	10+ (15-20)
Mean (SD)	3.4 (1.2)	3.8 (0.9)

*Available topics include benzodiazepine prescribing, chronic non-cancer pain, opioid use disorder, and opioid tapering.
