# Supplementary material for: Effects of an academic detailing service on benzodiazepine prescribing patterns in primary care
Source: PLoS One. 2023 Jul 27;18(7):e0289147. doi: 10.1371/journal.pone.0289147 (PMC10374092; doi:10.1371/journal.pone.0289147)
Supplement: S6 Table — (PDF) [file pone.0289147.s025.pdf]

S6 Table. Estimates of Percent Change in Slope of Long-Term Benzodiazepine Prescriptions After the Intervention vs Before

**Long-Term Prescriptions**	**Estimate (95% CI)**	**P-value**
**All Physicians**		
AD group	0.75 (0.17 to 1.32)	0.01*
Matched Controls	0.48 (0.16 to 0.81)	0.003*
% Difference (AD group vs Matched Controls)	0.26 (-0.40 to 0.92)	0.44
**Patients > 65**		
AD group	0.39 (-0.36 to 1.15)	0.31
Matched Controls	0.43 (0.03 to 0.83)	0.04*
% Difference (AD group vs Matched Controls)	-0.04 (-0.88 to 0.82)	0.93
**Top Prescribers**		
AD group	0.39 (-0.42 to 1.21)	0.34
Matched Controls	0.74 (0.04 to 1.44)	0.04*
% Difference (AD group vs Matched Controls)	-0.34 (-1.40 to 0.73)	0.53
