# Supplementary material for: Effects of an academic detailing service on benzodiazepine prescribing patterns in primary care
Source: PLoS One. 2023 Jul 27;18(7):e0289147. doi: 10.1371/journal.pone.0289147 (PMC10374092; doi:10.1371/journal.pone.0289147)
Supplement: S7 Table — (PDF) [file pone.0289147.s026.pdf]

S7 Table. Estimates of Percent Change in Slope of High-Risk Benzodiazepine Prescriptions After the Intervention vs Before

High-Risk Prescriptions	Estimate (95% CI)	P-value
**All Physicians**		
AD group	0.63 (-0.09 to 1.37)	0.09
Matched Controls	0.30 (-0.12 to 0.71)	0.16
% Difference (AD group vs Matched Controls)	0.34 (-0.50 to 1.18)	0.43
**Patients > 65**		
AD group	0.09 (-1.00 to 1.20)	0.87
Matched Controls	0.78 (0.19 to 1.37)	0.01*
% Difference (AD group vs Matched Controls)	-0.68 (-1.91 to 0.57)	0.28
**Top Prescribers**		
AD group	0.42 (-0.62 to 1.46)	0.43
Matched Controls	0.26 (-0.54 to 1.07)	0.52
% Difference (AD group vs Matched Controls)	0.15 (-1.15 to 1.48)	0.82
