# Supplementary material for: Effects of an academic detailing service on benzodiazepine prescribing patterns in primary care
Source: PLoS One. 2023 Jul 27;18(7):e0289147. doi: 10.1371/journal.pone.0289147 (PMC10374092; doi:10.1371/journal.pone.0289147)
Supplement: S8 Table — (PDF) [file pone.0289147.s027.pdf]

S8 Table. Estimates of Percent Change in Slope of New-Start Benzodiazepine Prescriptions After the Intervention vs Before

New Prescriptions	Estimate (95% CI)	P-value
**All Physicians**		
AD group	-0.58 (-1.60 to 0.46)	0.27
Matched Controls	-0.14 (-0.65 to 0.37)	0.58
% Difference (AD group vs Matched Controls)	-0.44 (-1.58 to 0.72)	0.46
**Patients > 65**		
AD group	-1.02 (-2.60 to 0.58)	0.21
Matched Controls	-0.72 (-1.56 to 0.13)	0.10
% Difference (AD group vs Matched Controls)	-0.31 (-2.11 to 1.52)	0.74
**Top Prescribers**		
AD group	0.00 (-1.55 to 1.57)	0.99
Matched Controls	-0.39 (-1.33 to 0.56)	0.42
% Difference (AD group vs Matched Controls)	0.38 (-1.44 to 2.24)	0.68
