# Supplementary material for: Effects of an academic detailing service on benzodiazepine prescribing patterns in primary care
Source: PLoS One. 2023 Jul 27;18(7):e0289147. doi: 10.1371/journal.pone.0289147 (PMC10374092; doi:10.1371/journal.pone.0289147)
Supplement: S9 Table — (PDF) [file pone.0289147.s028.pdf]

S9 Table. Estimates of Percent Change in Slope of Benzodiazepine-Related Patient Harms After the Intervention vs Before

**Patient Harms**	**Estimate (95% CI)**	**P-value**
**All Physicians**		
AD group	1.84 (-3.00 to 6.91)	0.46
Matched Controls	1.76 (-0.55 to 4.13)	0.14
% Difference (AD group vs Matched Controls)	0.07 (-5.17 to 5.61)	0.98
**Patients > 65**		
AD group	3.98 (-1.89 to 10.19)	0.19
Matched Controls	0.43 (-2.24 to 3.17)	0.75
% Difference (AD group vs Matched Controls)	3.53 (-2.89 to 10.37)	0.29
**Top Prescribers**		
AD group	-0.60 (-7.39 to 6.70)	0.87
Matched Controls	-1.56 (-6.01 to 3.09)	0.50
% Difference (AD group vs Matched Controls)	0.98 (-7.21 to 9.89)	0.82
