# Supplementary material for: Effects of an academic detailing service on benzodiazepine prescribing patterns in primary care
Source: PLoS One. 2023 Jul 27;18(7):e0289147. doi: 10.1371/journal.pone.0289147 (PMC10374092; doi:10.1371/journal.pone.0289147)
Supplement: S10 Table — (PDF) [file pone.0289147.s029.pdf]

S10 Table. Estimates of Percent Change in Slope After the Intervention vs Before – Sensitivity Analysis for Top Prescribing Physicians

**Total Prescriptions**		
AD group	-1.16 (-2.33 to 0.02)	0.05*
Matched Controls	-0.44 (-1.78 to 0.90)	0.52
% Difference (AD group vs Matched Controls)	-0.72 (-2.49 to 1.08)	0.43
**Long-Term Prescriptions**		
AD group	0.50 (-0.24 to 1.25)	0.19
Matched Controls	0.46 (-0.19 to 1.11)	0.17
% Difference (AD group vs Matched Controls)	0.04 (-0.94 to 1.03)	0.93
**High-Risk Prescriptions**		
AD group	-0.31 (-1.25 to 0.64)	0.52
Matched Controls	-0.28 (-1.09 to 0.54)	0.50
% Difference (AD group vs Matched Controls)	-0.03 (-1.28 to 1.23)	0.96
**New Prescriptions**		
AD group	1.04 (-0.83 to 2.94)	0.28
Matched Controls	-0.90 (-2.03 to 0.25)	0.13
% Difference (AD group vs Matched Controls)	1.95 (-0.27 to 4.21)	0.09
**Benzodiazepine-Related Harms**		
AD group	5.02 (-1.71 to 12.22)	0.15
Matched Controls	0.19 (-4.31 to 4.89)	0.94
% Difference (AD group vs Matched Controls)	4.83 (-3.29 to 13.63)	0.25
